# Subsets of extraocular motoneurons produce kinematically distinct saccades during hunting and exploration

**DOI:** 10.1016/j.cub.2024.12.010

**Published:** 2025-01-15

**Authors:** Charles K. Dowell, Thomas Hawkins, Isaac H. Bianco

**Affiliations:** 1 Department of Neuroscience, Physiology & Pharmacology, UCL, Gower Street, London WC1E 6BT, UK; 2 Department of Cell & Developmental Biology, UCL, Gower Street, London WC1E 6BT, UK

## Abstract

Animals construct diverse behavioral repertoires by moving a limited number of body parts with varied kinematics and patterns of coordination. There is evidence that distinct movements can be generated by changes in activity dynamics within a common pool of motoneurons or by selectively engaging specific subsets of motoneurons in a task-dependent manner. However, in most cases, we have an incomplete understanding of the patterns of motoneuron activity that generate distinct actions and of how upstream premotor circuits select and assemble such motor programs. In this study, we used two closely related but kinematically distinct types of saccadic eye movement in larval zebrafish as a model to examine circuit control of movement diversity. In contrast to the prevailing view of a final common pathway, we found that in the oculomotor nucleus, distinct subsets of motoneurons were engaged for each saccade type. This type-specific recruitment was topographically organized and aligned with ultrastructural differences in motoneuron morphology and afferent synaptic innervation. Medially located motoneurons were active for both saccade types, and circuit tracing revealed a type-agnostic premotor pathway that appears to control their recruitment. By contrast, a laterally located subset of motoneurons was specifically active for hunting-associated saccades and received premotor input from pretectal hunting command neurons. Our data support a model in which generalist and action-specific premotor pathways engage distinct subsets of motoneurons to elicit varied movements of the same body part that subserve distinct behavioral functions.

## Graphical abstract

**Graphical abstract:**
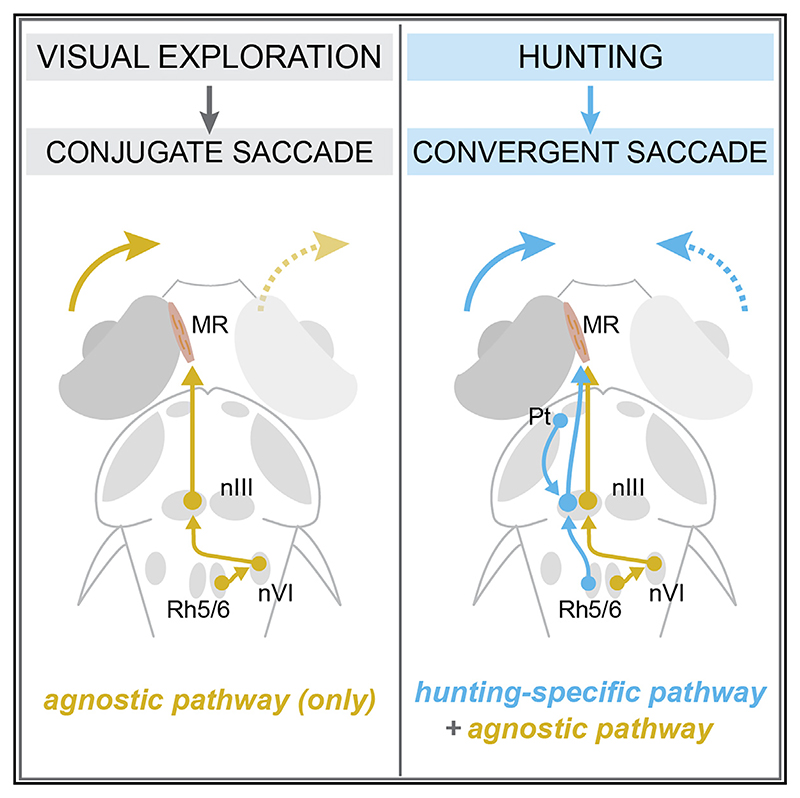

## Introduction

Animals move their individual body parts with varied kinematics and patterns of coordination to compose a broad variety of behaviors. This necessitates that different force profiles be generated by muscles, which in turn requires distinct patterns of motoneuron activity. Principles of motor control include size-ordered recruitment of motoneurons to produce increasingly forceful movements,^1^ force trajectories encoded by dynamic population activity,^2^ and task-specific recruitment.^3^ However, in most instances, we have an incomplete understanding of how movement diversity relates to population activity within motor pools and the circuit mechanisms by which premotor commands that encode specific kinematic variables or motor subroutines^4–7^ sculpt appropriate patterns of motoneuron activity.

The oculomotor system presents several advantages for elucidating how neural circuits generate a diverse motor repertoire. Several types of eye movement, with velocities spanning at least two orders of magnitude, support a variety of visual functions^8^ and are produced by only six eye muscles that are innervated by circumscribed pools of motoneurons in the brainstem. Recordings from these extraocular motoneurons have given credence to the idea of a “final common pathway,” in which different eye movement subsystems converge on a common population of motoneurons participating in all types of eye movement.^9^ However, this notion seems at odds with other anatomical and physiological evidence. Extraocular muscles are composed of diverse muscle fiber types, which are in turn innervated by motoneurons that vary in morphology, neurochemistry, physiological properties, and afferent inputs.^10^ Furthermore, physiological data have shown that the activity of single motoneurons can change in the context of distinct movement types^11,12^ and that motoneurons can be divided into subgroups with distinct dynamic properties.^13^ Such evidence suggests that motoneurons show at least some degree of selective activity and/or recruitment to produce eye movements with distinct kinematics or subserving different visuomotor functions.

**Figure 1:**
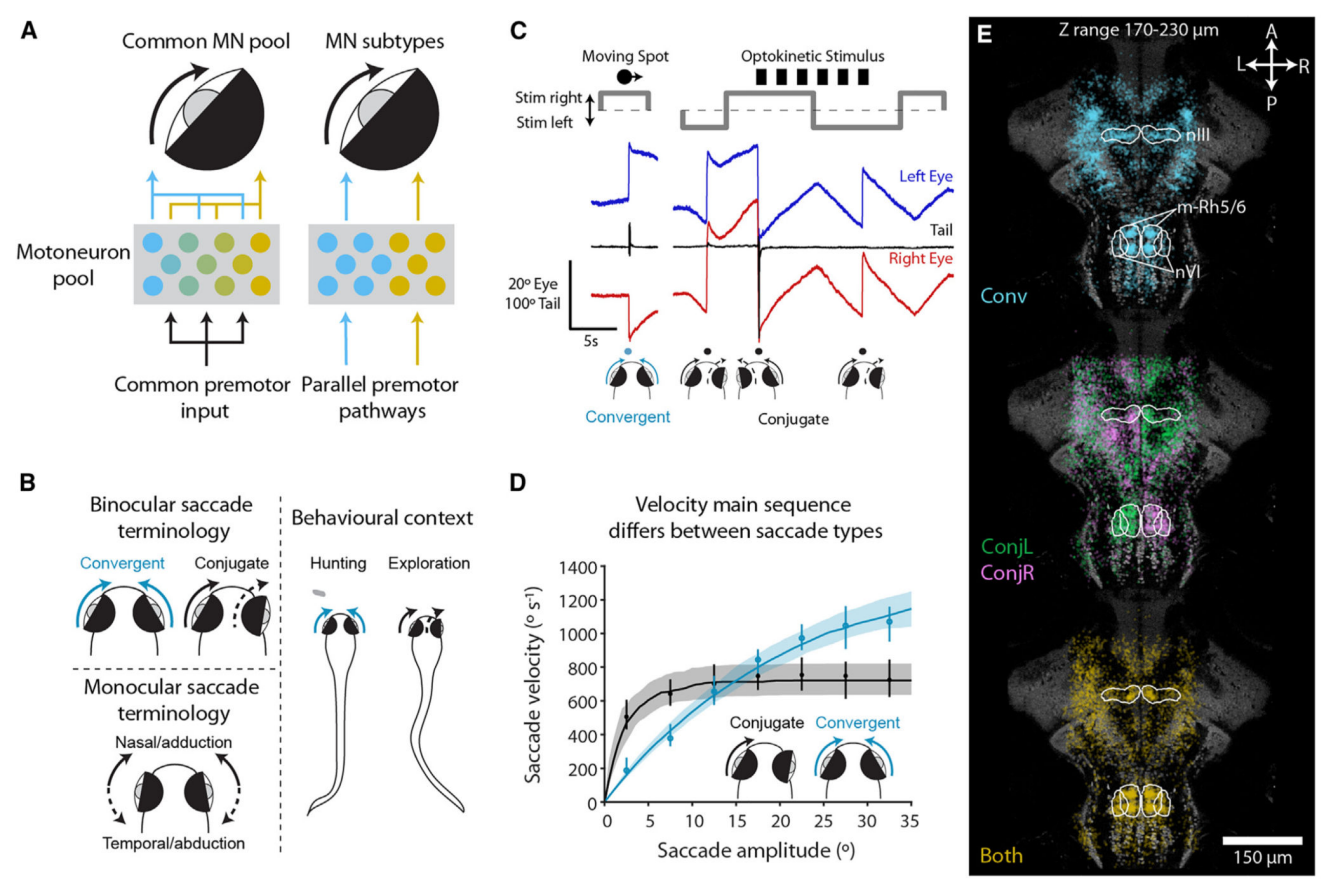
Saccade kinematics and oculomotor-tuned neurons (A) Schematic illustrating two models for neural control of distinct movements of the same plant (in this case the eye). Left: differences in spatiotemporal activity across a common population of motoneurons generates distinct muscle forces and eye kinematics. Right: task-specific subsets of motoneurons generate distinct actions. Note that models are not mutually exclusive. (B) Key to saccade terminology. (C) Example behavioral tracking during two-photon calcium imaging. Presentation of a prey-like moving spot evokes a hunting-related convergent saccade, and a drifting grating evokes conjugate saccades (OKR fast phases). (D) Velocity main sequence for convergent and conjugate adducting saccades. Line and shading show median ± IQR across exponential fits for *N* = 96 eyes. For reference, median (± IQR) velocity for each amplitude bin is shown for data pooled across animals. (E) Oculomotor-tuned ROIs active for convergent (*Conv*) or leftward/rightward conjugate (*ConjL/R*) or both (*Both*) saccade types. Images show a single focal plane in the mid/hindbrain. See also Figures S1 and S2 and Table S1.

To resolve this conundrum, we took advantage of two closely related but kinematically and ethologically distinct types of saccadic eye movement, which are expressed by larval zebrafish. Saccades are brief, extremely rapid eye movements, which enable animals to swiftly redirect gaze, and are generated by stereotypical “phasic-tonic” activity in extraocular motoneurons.^14,15^ Although it has been assumed that all horizontal saccades are controlled by a common neural pathway,^16^ we recently discovered that larval zebrafish generate different types of saccadic eye movement^17^ (Figure 1). Conjugate saccades are used to redirect gaze during exploration and recenter the eye during the opto-kinetic reflex, while convergent saccades play a specialized role during hunting to foveate prey targets and increase the extent and proximity of the binocular visual field.^17–20^ We found that adducting (nasally directed) horizontal saccades have distinct kine-matic properties in the binocular context of a conjugate saccade versus a convergent saccade,^17^ indicating that these two types of adducting saccades are generated by different patterns of extraocular motoneuron population activity.^21,22^

In this study, we leveraged these two saccade types to investigate two preeminent models for motor control: namely, whether distinct but related movements are generated by a shared pool of motoneurons or by action-specific subsets of motoneurons and how such activation patterns might be programed by premotor pathways (Figure 1A). Using cellular-resolution calcium imaging, we discovered topographically organized and saccade-type-specific activation of medial rectus motoneurons (MRMNs): medially located cells were active during both saccade types, whereas a laterally located subset was specifically engaged during convergent saccades. Electron microscopy (EM) revealed that this functional map aligned with three subtypes of MRMNs that differed in morphology and connectivity and suggested a synaptic mechanism for saccade-type-specific motoneuron activation. Specifically, medially located motoneurons appeared to obtain the majority of their synaptic input via a remarkable “giant synapse” from abducens internuclear neurons (INNs), and laser ablations confirmed that INNs were required for both types of adducting saccades. By contrast, laterally located motoneurons received a different compliment of synaptic inputs, including monosynaptic innervation from pretectal hunting command neurons, thus identifying a circuit motif that links sensorimotor decision-making to oculomotor output. In sum, our study supports a model in which parallel premotor pathways control the task-specific recruitment of subsets of motoneurons to generate kinematically distinct movements that subserve distinct ethological functions.

## Results

### Conjugate and convergent adducting saccades have distinct kinematics and associate with different patterns of brainstem activity

Here, we used the fact that zebrafish produce saccades with different kinematics during hunting versus during routine exploration to investigate how motoneurons generate distinct but related movements of the same body part (Figures 1A and 1B). To examine neuronal activity during behavior, we combined two-photon calcium imaging with high-speed recording of eye and tail movements (Figure 1C). Animals were shown prey-like moving spots to evoke hunting-related convergent saccades. In addition, drifting gratings were used to evoke the optokinetic response (OKR), which comprises “slow phase” eye rotations in the direction of whole-field motion and intermittent conjugate saccades (“fast phases”), which reset eye position when the eyes become eccentric in the orbit and display the same kine-matic properties as other conjugate saccades.^17^

Eye tracking confirmed that adducting saccades displayed distinct kinematics when they occurred in the binocular context of a convergent saccade, as compared with a conjugate saccade.^17^ We define convergent saccades as binocular events during which both eyes make an adducting (nasally directed) saccadic movement and conjugate saccades as events where both eyes rotate in the same direction (thus one eye adducts and the other abducts), but not necessarily with equal amplitudes (Figure 1B). Although the distribution of amplitudes and peak velocities was similar for convergent and conjugate adducting saccades (Figure S1A), when we examined the velocity “main sequence,” which is a characteristic feature of saccadic eye movements and relates peak velocity to saccade amplitude,^23^ we observed distinct relationships (Figure 1D). At small amplitudes, conjugate adducting saccades were faster, but velocity saturated at ~700°/s at amplitudes exceeding 10°. By contrast, convergent adducting saccades showed less saturation, and velocity continued to scale with amplitude. These observations were supported by statistically distinct exponential fits^24,25^ describing the main sequence relationships (AIC = 97.7%, *p* = 7.5 × 10^−15^, *N* = 86 eyes, signed-rank test versus single model). The eye also obtained more nasal post-saccadic positions following convergent saccades (convergent = 11.1° ± 0.2°, conjugate = 5.5° ± 0.2°, mean ± SEM across *N* = 152 eyes, *p* = 3.2 × 10^−50^, t test; Figure S1A). These observations and our previous characterization^17^ suggest that distinct patterns of motoneuron activity are responsible for controlling adducting saccades in these two behavioral contexts.

To identify neural activity associated with convergent and/or conjugate saccades, we performed calcium imaging in Tg (*elavl3*:H2B-GCaMP6s) transgenic animals, in which a genetically encoded calcium indicator is expressed broadly across the brain. In total, we recorded the activity of 1, 124, 129 automatically segmented regions of interest (ROIs; corresponding to individual neurons) across the midbrain and hindbrain of 76 animals and developed a two-stage analysis pipeline to identify ROIs that are putatively involved in generating saccadic eye movements (Figure S1; STAR Methods). In this way, we identified 44, 332 “oculomotor-tuned” ROIs (4.0% ± 0.2% of the total 1.1 _M_) and categorized them as active for either convergent (*Conv*) or conjugate (*Conj*) or both (*Both*) types of saccades. Brain volumes were registered to a reference brain (ZBB),^26^ allowing us to examine the anatomical distribution of oculomotor-tuned ROIs in a standard coordinate space (Figures 1E and S2). Of particular relevance to this study, oculomotor-tuned cells were enriched in the oculomotor nucleus (nIII) and abducens nucleus (nVI), a region of medial rhombomere-5/6 (m-Rh5/6) that has previously been shown to be active during ipsiversive conjugate saccades,^27–29^ and the pretectum adjacent to retinal arborization field 7 (AF7-Pt), which contains hunting command neurons.^30^

### Extraocular motoneurons are differentially recruited across saccade types

**Figure 2:**
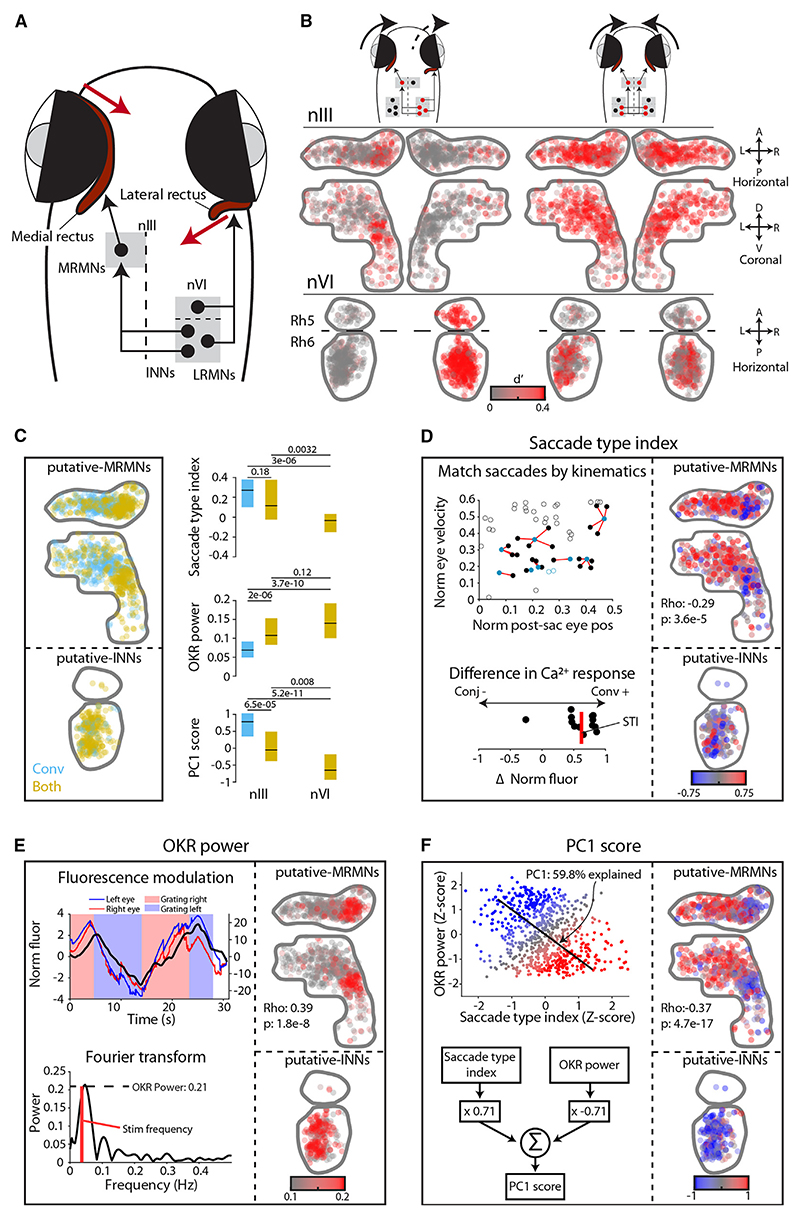
Subsets of extraocular motoneurons are engaged during conjugate versus convergent saccades (A) Horizontal gaze control by neurons in oculomotor (nIII) and abducens (nVI) nuclei. (B) Oculomotor-tuned ROIs colored by saccade-triggered activity (d′) for rightward conjugate saccades and convergent saccades (1,720 ROIs from 68 fish). (C) Oculomotor-tuned ROIs in nIII (pMRMNs, *N* = 538) and nVI (pINNs, *N* = 292) classified by saccade-type activation. In this and subsequent panels, right-sided ROIs have been reflected onto the left. Right: functional metrics (median with IQR) for pMRMNs and pINNs. *p* values from Kruskal-Wallis with Dunn-Sidak post hoc test. (D) Saccade-type index. Left: process of computing saccade-type index for an example neuron. First, each convergent saccade (blue) is matched to a conjugate saccade (black) having similar peak velocity and post-saccadic eye position. Matched pairs are indicated with red lines. The difference in saccade-triggered fluorescence modulation is then computed for each matched pair (black dots in lower plot), and saccade-type index for the cell is the median of these measures across all matched pairs (red line). Right: pMRMNs in nIII and pINNs in nVI, color coded by saccade-type index (*N* = 743 ROIs). (E) OKR power. Left: process of computing OKR power, illustrated for an example cell. First, median fluorescence modulation is computed during optokinetic stimulation. Then the power spectrum of the fluorescence signal is computed, and OKR power is measured at the stimulus frequency (red line). Right: as per (D), but for OKR power (*N* = 830 ROIs). (F) PC1 score. Left: illustration of PC1. Right: as per (D), but for PC1 score (*N* = 743 ROIs). See also Figure S3.

Saccades are generated by phasic-tonic activity in extraocular motoneurons, where a brief “pulse” of spiking rapidly accelerates the eye and firing rate then declines to a tonic level to hold the eye in its new position.^14^ Our velocity main sequence analysis suggests that the pulse component, which controls eye velocity, saturates for conjugate adducting saccades but continues to scale with amplitude during convergent saccades. This might be due to differences in net population activity within a common pool of motoneurons or result from recruitment of different subsets of motoneurons between saccade types. We therefore examined activity in the nIII and nVI, which contain MRMNs, lateral rectus motoneurons (LRMNs), and INNs, which have well-established roles in generating horizontal eye movements (Figure 2A).

Oculomotor-tuned cells showed distinct patterns of saccade-triggered activity modulation (d′) during conjugate versus convergent saccades (Figure 2B). For conjugate saccades, we observed the expected pattern of lateralized activity, consistent with unilateral activity in MRMNs (ipsilateral to the adducting eye) as well as LRMNs and INNs in the contralateral nVI (i.e., ipsilateral to the abducting eye). By contrast, convergent saccades showed symmetric activity, consistent with bilateral activation of INNs driving bilateral activation of MRMNs to produce adduction of both eyes. The identities of MRMNs, INNs, and LRMNs were supported by eye-direction tuning of individual cells. By fitting rectilinear functions relating saccade-triggered fluorescence to post-saccadic eye position (Figure S3A), we found that in nVI, *Both* ROIs were tuned to adduction of the contralateral eye, identifying these cells as putative INNs (pINNs), whereas *Conj* ROIs were activated for abduction of the ipsilateral eye, as expected for LRMNs (Figures S3B and S3C). In nIII, *Both* and *Conv* ROIs were tuned to adduction of the ipsilateral eye, as expected for MRMNs,^31^ and are thus designated putative MRMNs (pMRMNs). Moreover, these cells were located in dorsal nIII, where retrograde tracing from eye muscles has localized MRMNs in larval zebrafish.^32^ *Conj* ROIs in nIII occupied scattered locations and showed no systematic nasal/temporal preference (Figure S3B) and are not considered further.

In support of the hypothesis that recruitment of distinct sub-sets of motoneurons might underlie distinct saccade types, we observed strikingly different patterns of pMRMN activity during conjugate versus convergent saccades. For conjugate saccades, activity was restricted to a compact dorso-medial region, whereas for convergent saccades, activity extended much more broadly across dorso-lateral nIII (Figure 2B). Very similar patterns were observed when saccades were binned according to amplitude or post-saccadic eye position (Figure S3D), supporting the idea that these differences are a consistent function of saccade *type*. This pattern could also be seen in maps of ROIs labeled by their saccade-type recruitment (Figure 2C): *Both* ROIs were restricted to dorso-medial nIII, but *Conv* ROIs extended across a broader dorso-lateral region. In sum, these results are consistent with differential recruitment of MRMNs generating convergent versus conjugate adducting saccades.

### Functional topography within nIII

Next, we analyzed the response properties of individual neurons, which provided further support for saccade-type-specific activity and revealed a topographic organization of functional properties across the pMRMN population.

To assess the extent to which a cell’s activity was specifically modulated as a function of saccade type, independent of its eye position or velocity sensitivity, we developed a metric we termed “saccade-type index.” Specifically, we computed the median difference in saccade-triggered calcium response between conjugate versus convergent saccades, which were pairwise matched for similar post-saccadic eye positions and peak velocities (Figure 2D). A positive index indicates a greater calcium response for convergent saccades, whereas a negative index indicates a greater response during conjugate saccades. Maps of pMRMNs coded by saccade-type index revealed a clear topography within nIII (Figure 2D). In dorso-medial nIII, the mean index was close to zero, indicating no overall preference for either saccade type. However, in dorso-lateral nIII, most cells had a positive index, indicating greater activity during convergent saccades. This pattern mirrored the anatomical segregation of *Both* and *Conv* ROIs (Figure 2C) and supports the notion that individual motoneurons are differentially active as a function of saccade type.

Functional topography was further evidenced by an independent functional metric, “OKR power,” which quantified the modulation of single-neuron fluorescence during slow phase eye movements (Figure 2E; STAR Methods). To provide a compact summary of single-cell activity, we used principal-component analysis to compute a weighted sum of saccade-type index and OKR power (Figure 2F). The resulting “PC1 score” increased significantly across the medial→lateral axis of nIII, and consequently, laterally located *Conv* pMRMNs had significantly higher scores than medially localized *Both* pMRMNs (Figures 2C and 2F). In nVI, *both* ROIs (pINNs, see above) had low PC1 scores that were most similar to *Both* pMRMNs, suggesting INNs might comprise their dominant afferent input.

In summary, our data support a model in which INNs recruit MRMNs in dorso-medial nIII during both types of adducting saccades, but during convergent saccades, additional MRMNs are recruited in dorso-lateral nIII.

### Three subtypes of MRMNs with distinct morphology and afferent connectivity

We next asked if there were structural correlates of these functional differences by examining neuronal morphology and synaptic connectivity.

**Figure 3:**
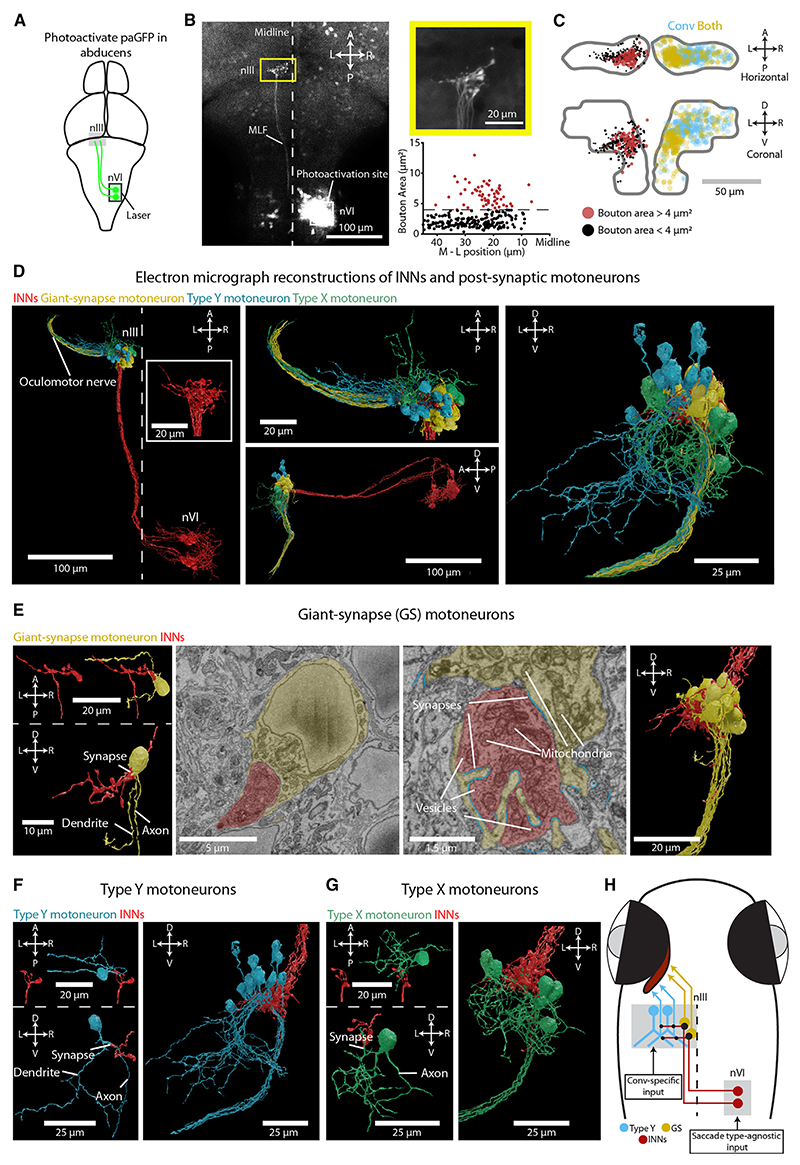
Three subtypes of medial rectus motoneuron (A) Schematic of paGFP photoactivation experiment with 40×40×20 – 30 μm target volume in nVI. (B) Left: example paGFP labeling of INN projections from abducens to oculomotor nucleus. Right, top: high magnification image of INN axon terminals. Right, bottom: cross-sectional area of axonal boutons versus mediolateral position in nIII (269 boutons from 7 animals). (C) Bouton locations in left nIII and oculomotor-tuned ROIs (shown on right). Circles indicate bouton locations and are scaled according to cross-sectional area. (D) Ultrastructural reconstructions of 15 INNs along with 35 postsynaptic motoneurons. Midline indicated by dashed line. (E) Giant-synapse motoneurons. Left: 3D renderings of an INN terminal arbor and postsynaptic giant-synapse motoneuron. Middle: Electron micrographs of the axon terminal (red) and postsynaptic motoneuron (yellow). Automatically detected synapses shown by blue lines. Right: 3D rendering of all 15 giant-synapse motoneuron somata, each associated with one INN. (F and G) 3D reconstructions of single (left) and all (right) type Y (F, 12 cells) and type X (G, 6 cells) motoneurons. (H) Circuit model (see text for explanation). See also Figures S4 and S5.

We started by examining INNs, as these neurons make excitatory, monosynaptic connections onto MRMNs,^33–37^ and our imaging data indicate that they are active for both types of saccades. First, we visualized INN projections by performing two-photon photoactivation of paGFP in nVI of Tg(Cau.Tuba1:c3paGFP) a7437; Tg(elavl3:jRCaMP1a)jf16 double-transgenic animals (Figure 3A). As expected,^38,39^ axons of photolabeled INNs crossed the ventral midline at the level of nVI and ascended in the contralateral medial longitudinal fasciculus before arborizing in nIII (Figure 3B). We noticed that INN axonal boutons in the nIII varied in size (Figure 3B), with particularly large boutons (≥ 4 μm^2^ cross-sectional area) occupying a medial region that corresponded to the location of *Both* pMRMNs (Figure 3C) while smaller boutons were distributed over a broader mediolateral extent.

This observation led us to hypothesize that distinct patterns of synaptic connectivity between INNs and MRMNs might accompany the topographic gradient of motoneuron functional properties. To investigate, we used a whole-brain serial-blockface scanning electron micrography dataset^40^ to trace 15 INNs in one hemisphere, along with 35 postsynaptic neurons (Figure 3D). All of the postsynaptic neurons extended an axon in the third cranial nerve, and because they also receive input from INNs, they are assumed to be MRMNs. Ultrastructural reconstructions revealed that individual INNs had multiple synaptic boutons within nIII. Remarkably, every INN established a giant synapse with a single MRMN (*N* = 15) (Figures 3E and S4A). This synapse was formed between one especially large bouton and claw-like invaginations of the soma of the postsynaptic motoneuron. To our knowledge, this synapse has not previously been described in any species. To better resolve the synaptic contacts, we obtained additional transmission electron micrographs, which revealed multiple postsynaptic densities at the apposition between large axon terminals and the soma of putative motoneurons (Figure S5), indicating that these giant synapses contain multiple sites of neurotransmission. The postsynaptic MRMNs, which we refer to as giant synapse (GS) motoneurons, otherwise had small and simple dendritic trees (Figures 3E and S4A), suggesting that the majority of their synaptic input derives from the giant synapse in a one-to-one connectivity motif with an INN. We also reconstructed two motoneurons with simple dendritic arbors that formed large claw-like postsynaptic contacts with multiple INN boutons and had somata that sat adjacent to the other GS MRMNs (Figure S4B).

In addition to the giant synaptic terminal, all INNs additionally had smaller boutons that contacted MRMNs that we classified into two types based on morphology: type X and type Y. Type Y motoneurons had large, ventrally directed dendritic arbors with prominent Y-shaped branches (*N* = 12, Figures 3F and S4C; plus *N* = 5 cells in the opposite brain hemisphere, Figure S4E). INNs did not synapse onto these distal dendrites but rather onto the axons or proximal dendrites of the cells (Figures 3F and S4C). Type X motoneurons (*N* = 6) elaborated dendritic arbors in medial and anterior portions of nIII, and INNs synapsed onto their somata or distal dendrites (Figures 3G and S4D).

The cell bodies of GS, type Y, and type X MRMNs occupied distinct positions within the nIII (Figure 3D), and the distribution of GS and type Y motoneurons in particular bore striking resemblance to the mediolateral functional topography of pMRMNs (compare Figures 3C and 3D). GS motoneurons occupied dorso-medial locations, similar to *Both* pMRMNs that were active for conjugate and convergent saccades and have PC1 scores similar to INNs. By contrast, type Y motoneurons were located dorso-laterally, likely corresponding to pMRMNs that displayed positive saccade-type indices and that were selectively recruited during convergent saccades.

Together, these observations support a model in which topographically organized synaptic connectivity contributes to distinct functional properties and saccade-type-specific recruitment of MRMNs (Figure 3H). In this model, the giant synapses between INNs and medially located GS motoneurons provide a strong feedforward relay of INN activity such that both cell types share similar functional profiles and are active for both types of saccades. Type Y motoneurons also receive excitatory input from INNs on their axons and proximal dendrites, but they likely receive additional afferent input that underlies the convergent saccade-specific activity observed in lateral pMRMNs.

### INNs are necessary for both convergent and conjugate adducting saccades

Our model predicts that INNs provide a significant synaptic input to MRMNs for the production of both conjugate and convergent adducting saccades. To test the necessity for INN innervation, we performed two loss-of-function experiments designed to abrogate INN input.

**Figure 4:**
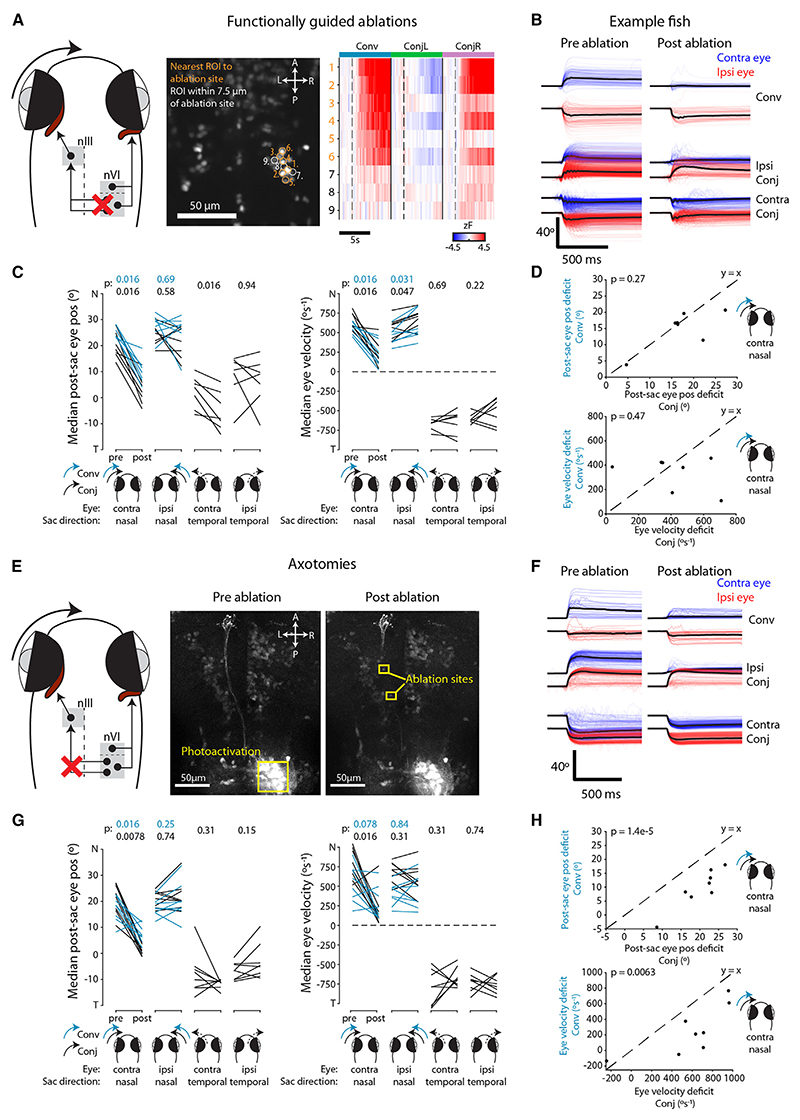
Abducens internuclear neurons are necessary for both conjugate and convergent adducting saccades (A) Left: schematic of ablations. Right: example plane showing locations of functionally targeted ROIs and their median saccade-triggered activity. Dashed line indicates saccade time, and *zF* is normalized calcium fluorescence (see STAR Methods). (B) Eye position traces for saccades pre- and post-ablation, for an example fish. Black lines show median across trials. *Ipsi* and *Contra* refer to eye (ipsilateral or contralateral) and saccade direction (ipsiversive or contraversive), with respect to the ablation site. (C) Post-saccadic eye position and eye velocity for conjugate and convergent saccades, pre- and post-ablation. Median across saccades for each of *N* = 7 animals. (D) Deficits in post-saccadic eye position (top) and velocity (bottom) for conjugate versus convergent nasal saccades of the contralateral eye. Deficits computed as differences between medians pre- and post-ablation. (E) Left: schematic of axotomies. Right: example axotomy of photolabeled INN axons in the medial longitudinal fasciculus (MLF). (F–H) As per (B)–(D), but for axotomies (*N* = 8 animals). *p* values in (C) and (G) are for signed-rank tests and in (D) and (H) for t tests.

First, we used a pulsed infrared laser to ablate the somata of functionally identified INNs (Figure 4A). To do this, we first used two-photon calcium imaging in nVI to identify neurons that were active during convergent saccades, reasoning that these cells should correspond to INNs (rather than LRMNs; see above). After ablation of functionally identified pINNs (14 ± 3 cells in *N* = 7 animals), we observed a substantial impairment in post-saccadic position and velocity of the contralateral eye during both conjugate and convergent adducting saccades (Figures 4B–4D). The contralateral eye also obtained a more eccentric position following temporal saccades, likely as a result of reduced tone in MRMNs. Finally, we observed a small increase in nasal velocity of the ipsilateral eye, perhaps due to unintended damage to LRMNs (Figure 4C).

Second, we performed laser axotomies of INN projections in the medial longitudinal fasciculus, which we targeted following photolabeling with paGFP (*N* = 8 animals) (Figure 4E). Axotomy caused similar deficits in adducting saccades of the contralateral eye (Figures 4F and 4G), although the deficit was weaker for convergent saccades (Figure 4H). In part, we suspect this is due to the ablations failing to cut all INN axons and conjugate adducting saccades having a greater dependence on INN innervation.

In summary, loss-of-function experiments support a model in which INN input to MRMNs is required for both convergent and conjugate adducting saccades with normal kinematics. The lesser impact of axotomies on convergent saccades is in line with our hypothesis that additional afferent input is involved in generating this saccade type.

### m-Rh5/6 makes functionally distinct connections to INNs and type Y motoneurons

We next sought to identify premotor input to INNs, which our model predicts would be involved in generating both saccade types, as well as parallel inputs to type Y motoneurons that should play a specific role in convergent adducting saccades. Horizontal saccades are triggered by disinhibition of burst neurons in the paramedian pontine reticular formation (PRF) and medullary reticular formation, which in turn provide eye velocity signals to nVI.^14^ Because saccadic burst neurons have been optogenetically mapped to rhombomere 5 in larval zebrafish,^41^ we focused on m-Rh5/6, where we observed a concentration of neurons that were active during convergent saccades (*Conv*) or both saccade types (*Both*) (Figures 1 and S2).

**Figure 5:**
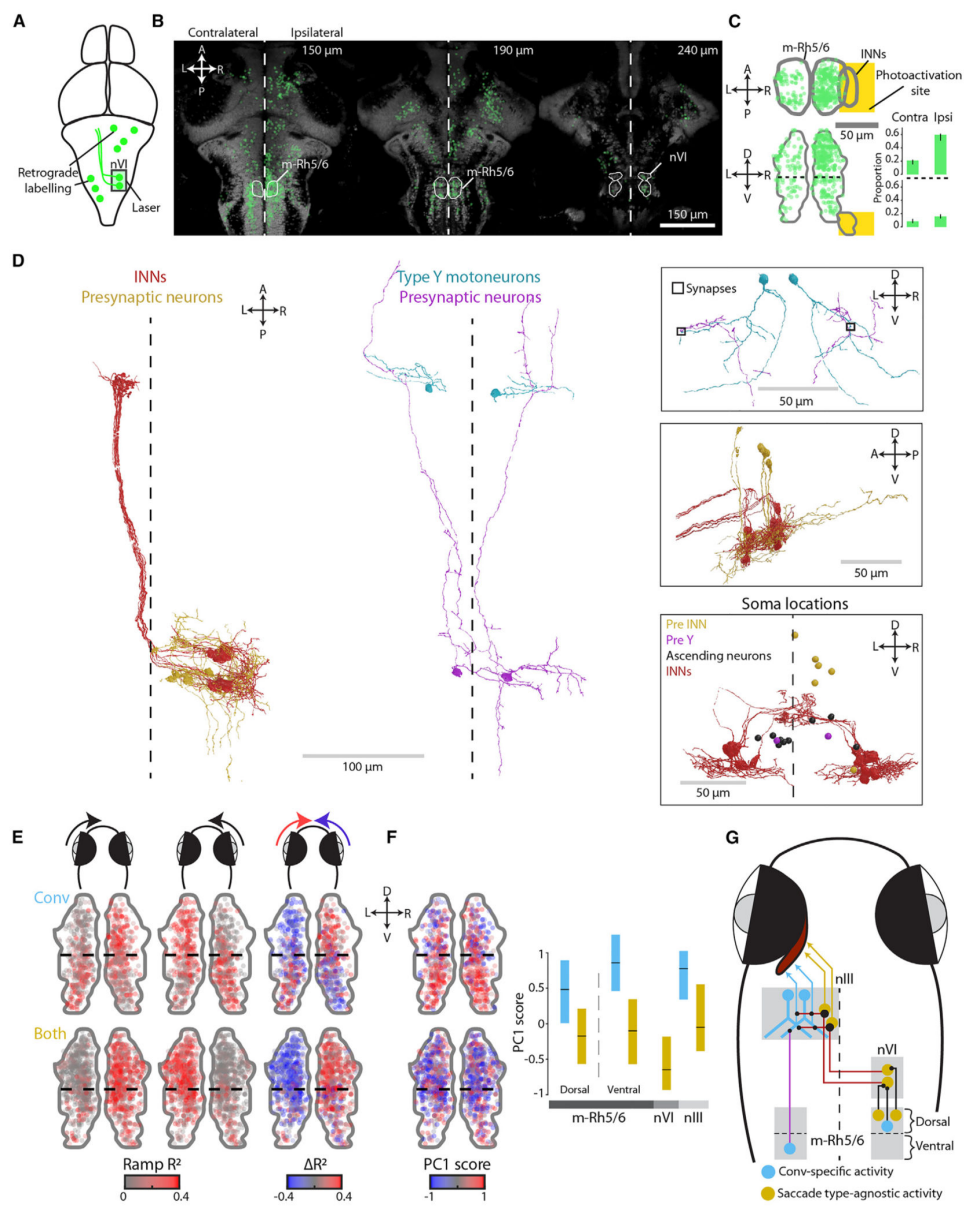
m-Rh5/6 makes functionally distinct connections to INNs and type Y motoneurons (A) Schematic of retrograde photolabeling from abducens. (B) Retrogradely labeled somata plotted on horizontal planes in ZBB reference space (4,063 neurons from 7 animals). (C) Locations of retrogradely labeled neurons in m-Rh5/6 (440 neurons). Inset shows proportion of cells labeled in dorsal and ventral domains of m-Rh5/6, ipsilateral and contralateral to photoactivation site (median [IQR], *N* = 7 animals). (D) Ultrastructural reconstructions of six neurons in m-Rh5/6 that are presynaptic to INNs (left) and two cells that are presynaptic to type Y motoneurons (right). Inset boxes show synapses, alternate views, and soma locations of other ascending neurons from m-Rh5/6. (G) Topography in eye-direction tuning along dorso-ventral axis of m-Rh5/6 (891 *Conv*, 1,447 *Both* ROIs from 74 fish). Left, middle: rectilinear fit *R*^2^ (see STAR Methods and Figure S3A) for nasal rotation of the left and right eye. Right: cell-wise difference in *R*^2^ for left versus right eye nasal rectilinear fits. There is a switch in tuning from contralateral to ipsilateral eye along the dorsal to ventral axis. (H) PC1 score for oculomotor-tuned ROIs in m-Rh5/6 (814 *Conv*, 1,299 *Both* ROIs from 71 fish). Plot shows median (IQR) PC1 score across ROIs in m-Rh5/6, as well as pINNs and pMRMNs (from Figure 2) for comparison. (I) Circuit model (see text for explanation). See also Figure S6.

Neurons located in the dorsal part of m-Rh5/6 provided direct synaptic input to ipsilateral INNs. We showed this by first photo-activating paGFP in nVI and subsequently identifying retrogradely labeled cell bodies (Figures 5A and 5B). Photolabeled somata were observed in various mid/hindbrain regions, including a high density of cells in the dorsal region of m-Rh5/6, ipsilateral to the photoactivation site (Figure 5C). To verify this putative connection, we reconstructed cells from the Svara et al.^40^ EM dataset. By tracing cells from their presynaptic terminals with INNs back toward their somata, we identified five neurons in dorsal m-Rh5/6 (Figure 5D), thus confirming a monosynaptic connection onto INNs.

Ultrastructural data also revealed that neurons in ventral m-Rh5/6 provide afferent input to type Y motoneurons. We showed this by reconstructing cells with somata in ventral m-Rh5/6 and in so doing identified 11 neurons with ipsilateral ascending projections to the caudal midbrain (Figures 5D and S6A). For two of these neurons, we confirmed synaptic connections onto the distal Y-shaped dendrites of type Y motoneurons (Figure 5D), thereby identifying an INN-independent afferent input to this subtype of MRMN. We also identified 11 additional neurons, in diverse mid/hindbrain locations, that were presynaptic to type Y motoneurons (Figure S6B), indicating that they receive several sources of innervation.

Based on these findings that dorsal m-Rh5/6 innervates INNs and ventral m-Rh5/6 innervates type Y motoneurons, we hypothesized that there should be a dorso-ventral topography of functional properties in m-Rh5/6. Indeed, when we fit rectilinear functions to oculomotor-tuned ROIs, we observed a switch in eye-direction tuning along the dorso-ventral axis (Figure 5E): dorsal cells were tuned to adduction of the contralateral eye, consistent with ipsilateral input to INNs (which in turn innervate contralateral MRMNs), whereas *Conv* ROIs in ventral m-Rh5/6 were tuned to adduction of the ipsilateral eye, consistent with projections to ipsilateral type Y MRMNs. In addition, OKR power decreased, and saccade-type index increased from dorsal to ventral m-Rh5/6 (Figure S6C). As a result, *Conv* ROIs in ventral m-Rh5/6 had high PC1 scores and thus appeared functionally similar to *Conv* pMRMNs in dorso-lateral nIII. By contrast, *Both* ROIs, especially in dorsal m-Rh5/6, had lower PC1 scores, similar to pINNs and *Both* pMRMNs in dorso-medial nIII (Figure 5F).

Together, these data support a model in which m-Rh5/6 contributes to separate premotor channels to recruit the subsets of motoneurons that produce conjugate and convergent adducting saccades (Figure 5G). Cells in dorsal m-Rh5/6 are putative saccadic burst neurons that innervate INNs to generate both types of saccades. By contrast, neurons in ventral m-Rh5/6 make direct connections onto type Y motoneurons and appear to provide parallel, convergent saccade-specific signals.

### m-Rh5/6 is required for adducting saccades, and its activation drives nasal eye movement

We next performed gain- and loss-of-function experiments to probe a causal role for m-Rh5/6 in the generation of saccadic eye movements.

**Figure 6:**
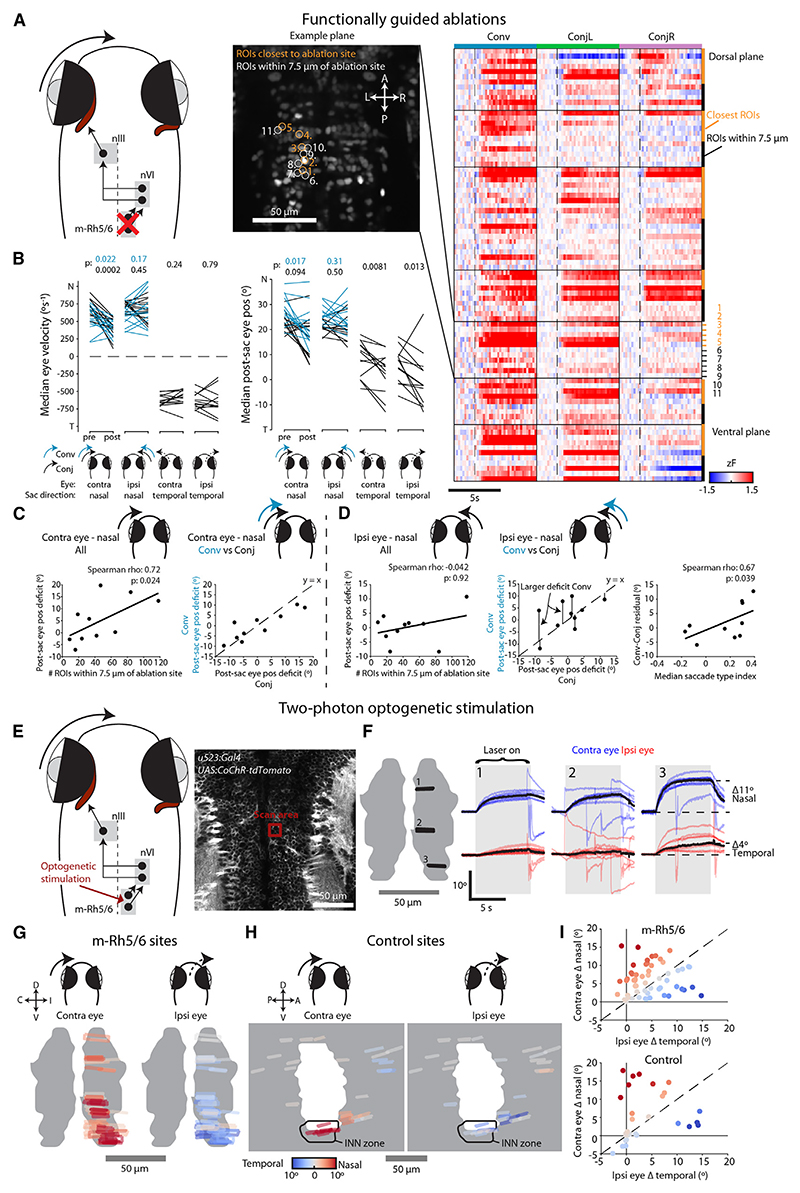
Ablation and optogenetic activation support a role for m-Rh5/6 in control of adducting saccades (A) Left: schematic of ablations. Right: functionally targeted ROIs from one example focal plane and saccade-triggered activity for all targeted ROIs across seven planes in the same animal. (B) Eye velocity and post-saccadic eye position pre- and post-ablation (medians across saccades for each of *N* = 13 animals, signed-rank test). (C) Post-saccadic eye position deficit for adducting saccades of the eye contralateral to ablation site. Left: deficit versus number of ROIs within 7.5 μm of ablation site. Right: comparison of deficit for convergent versus conjugate saccades. (D) Post-saccadic eye position deficit for adducting saccades of the eye ipsilateral to ablation site. Left, middle: as per (C). Right: difference between deficit for convergent and conjugate saccades (Conv-Conj residual) versus median saccade-type index of ablated cells. (E) Schematic of two-photon optogenetic stimulation of m-Rh5/6 neurons and example scan site. (F) Eye position traces from an example animal for stimulation of three sites (indicated on a frontal view of m-Rh5/6 mask). Black lines show medians across trials. (G and H) Stimulation sites (G; *N* = 60 from 7 fish) and control sites (H; *N* = 36 from 8 fish), color coded by change in position of the contralateral (left) and ipsilateral (right) eye. All sites registered to ZBB reference space and depicted on the right hemisphere. (G) Pairwise comparison of change in position of the contralateral versus ipsilateral eye. Points colored according to distance from *y* = *x* line (dashed diagonal).

First, we performed functionally guided laser ablations (Figure 6A). Removal of saccade-active m-Rh5/6 neurons resulted in eye velocity deficits during adducting saccades of the contralateral eye (Figure 6B), similar to ablations of INNs. Deficits in post-saccadic position scaled with the number of ablated neurons and were very similar for conjugate and convergent saccades (Figure 6C). The effect of ablations on the ipsilateral eye was more complex, producing less robust velocity and position deficits (Figure 6B). Moreover, the effect on post-saccadic eye position showed substantial variability between saccade types (Figure 6D). To attempt to explain this variability, we analyzed the functional properties of ablated neurons and observed that the difference in effect size between convergent versus conjugate saccades (“Conv-Conj residual”) was linearly related to the median saccade-type index of the ablated neurons (Figure 6D). Together, these data support a model in which m-Rh5/6 input to ipsilateral INNs is required to drive both types of adducting saccades in the contralateral eye. Although adducting saccades of the ipsilateral eye were less impacted, the greater sensitivity of convergent saccades to loss of neurons with high saccade-type index is in line with a convergent-specific ipsilateral input onto type Y motoneurons.

Next, we used multiphoton optogenetics to show that activating m-Rh5/6 neurons was sufficient to evoke nasal eye rotations. Specifically, we used Tg(*u523*:Gal4);Tg(UAS:CoChR-tdTomato) transgenic animals, in which the excitatory opsin CoChR is broadly expressed in mid/hindbrain, and performed two-photon photostimulation at localized sites within m-Rh5/6 (Figure 6E). All sites evoked adduction of the contralateral eye, albeit to variable extents, compatible with m-Rh5/6 innervating INNs (Figures 6F and 6G). By contrast, control stimulations in loci surrounding m-Rh5/6 produced little eye movement, except for stimulation in the region of INN somata which, as expected, evoked robust adduction of the contralateral eye (Figure 6H). Most photostimulations in m-Rh5/6 also evoked abducting movements of the ipsilateral eye. Moreover, across different sites, we observed independent variation in the effects on the contralateral versus ipsilateral eye, suggesting a largely monocular organization of nIII commands within m-Rh5/6 (Figure 6I).

In sum, anatomical, functional imaging and gain- and loss-of-function experiments support the existence of two output pathways from m-Rh5/6. Dorsal m-Rh5/6 innervates INNs, and this pathway appears both necessary and sufficient for adducting saccades of the contralateral eye. Ventral m-Rh5/6 contains neurons that directly innervate ipsilateral type Y motoneurons. However, the weaker effects of ablation and optogenetic stimulation on adduction of the ipsilateral eye seem compatible with this pathway operating in parallel to INN input and being only one of several potential sources of convergent saccade-specific innervation of motoneurons.

### Pretectal command neurons make synaptic connections onto oculomotor targets

Zebrafish use conjugate saccades to shift gaze during routine exploration of their environment and to recenter the eye during OKR, whereas convergent saccades are deployed during hunting to binocularly foveate prey.^17^ How do descending commands interface with oculomotor circuits to generate appropriate saccade types in distinct ethological contexts?

**Figure 7:**
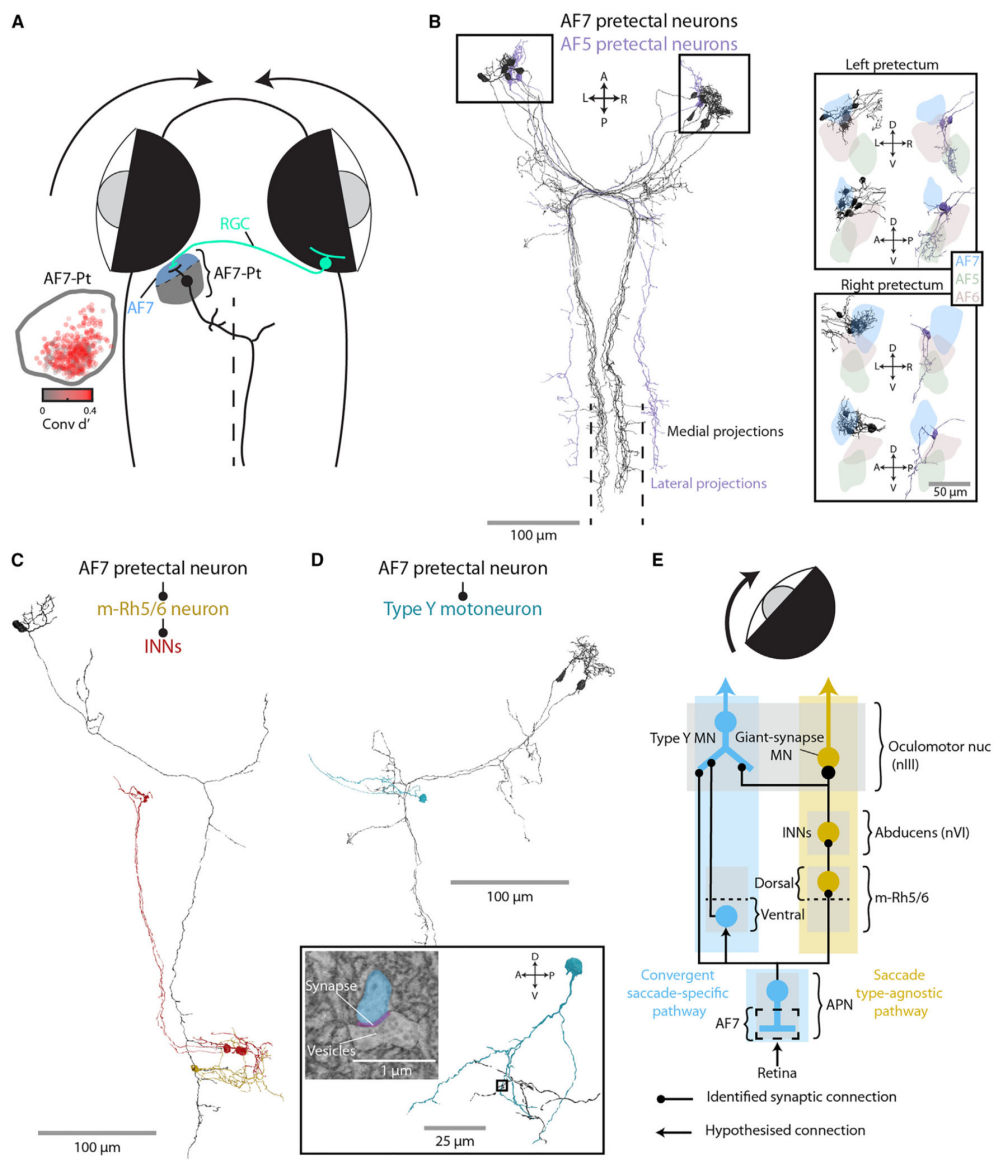
Pretectal projection neurons and overall circuit model (A) Schematic of retinal arborization field 7 (AF7), adjacent pretectum (AF7-Pt), and an anterior pretectal nucleus (APN) command neuron (black). Inset: convergent saccade-triggered activity modulation (Conv d′) for oculomotor-tuned ROIs in AF7-Pt (485 neurons from 30 fish). (B) Ultrastructural reconstructions of pretectal projection neurons (12 AF7-pretectal cells and 3 AF5-pretectal cells). Main panel shows horizontal projection, and inset boxes show coronal and sagittal views of dendritic arbors relative to retinal arborization fields (AF5–7). (C) Horizontal projection showing connectivity from an AF7-pretectal neuron to postsynaptic cells in m-Rh5/6 and then to abducens INNs. (D) Two AF7-pretectal neurons and postsynaptic type Y motoneuron (caudal extent of the pretectal axons has been cropped). Inset box shows an electron micrograph and location of one of the synapses. (E) Circuit model. Two types of saccades are controlled by parallel premotor pathways that activate subsets of motoneurons: a saccade-type-agnostic pathway (yellow) and a convergent saccade-specific pathway (blue). Both conjugate and convergent adducting saccades involve activation of the type-agnostic pathway in which INNs receive input from dorsal m-Rh5/6 and in turn provide giant-synaptic input to GS MRMNs in dorso-medial nIII. In the convergent saccade-specific pathway, type Y MRMNs in dorso-lateral nIII are additionally recruited, likely by a combination of INN, ventral m-Rh5/6, and pretectal innervation. Descending commands from APN command neurons activate both pathways to produce convergent saccades in the context of hunting. See also Figure S7.

We previously identified neurons in the anterior pretectal nucleus (APN), which are labeled by the *KalTA4u508* transgene and function as a command system to induce hunting routines.^30^ Hunting invariably commences with a convergent saccade,^18^ and accordingly, we observed many ROIs in this region of pretectum, adjacent to retinal AF7, which showed substantial convergent saccade-triggered modulation of calcium fluorescence (Figure 7A). Because optogenetic activation of APN command neurons is sufficient to trigger convergent saccades—in addition to other motor components of hunting routines—and a subset of these cells project to the mid/hindbrain,^30^ we investigated whether they might contact any of the circuit elements involved in convergent saccade generation.

Indeed, ultrastructural reconstructions revealed that AF7-pretectal neurons made synaptic connections with targets in m-Rh5/6 as well as type Y MRMNs. We examined 12 AF7-pretectal neurons from the Svara et al.^40^ EM dataset whose soma locations and axo-dendritic morphologies matched those described for *KalTA4u508* APN command neurons^30^ (Figure 7B). These neurons extended dendrites into AF7 (Figure 7B, inset) and projected long axons that decussated in the vicinity of nIII and then extended caudally in the contralateral hindbrain reticular formation, very close to the midline (~ 5 μm from the midline). We identified synaptic connections between the axons of these putative APN command neurons and cells in m-Rh5/6 (Figure S7B), with one postsynaptic neuron itself contacting two INNs (Figure 7C). Furthermore, we identified synaptic connections from two AF7-pretectal neurons onto the distal dendrite of a contralateral type Y motoneuron (Figure 7D), indicating that descending pretectal commands directly impinge on the motoneurons that we suggest are preferentially active during convergent saccades.

We additionally reconstructed a second type of pretectal neuron, whose afferent and efferent connectivity suggested a role in conjugate eye movements. These neurons (*N* = 3) extended dendrites into retinal AF5, which contains the axon terminals of direction-selective retinal ganglion cells^42^ and projected axons that decussated near nIII and coursed through the contralateral tegmentum at a lateral position, ~ 50 μm from the midline (Figure 7B). We identified synaptic connections from these AF5-pretectal neurons onto the somata of both INNs and LRMNs as well as neurons in dorsal m-Rh5/6 (Figure S7A). Neurons in this region of the larval zebrafish pretectum are believed to play a similar role to the mammalian accessory optic system (DTN/NOT) and to process whole-field visual motion to control the OKR and optomotor response.^42–44^ To our knowledge, there is currently little evidence for direct connections from this region of pretectum to the nVI in any species,^45,46^ but the morphology and connectivity of these AF5-pretectal projection neurons appear well suited to control conjugate eye movements in response to whole-field motion.

In sum, we identified two types of pretectal projection neurons with distinct afferent inputs and efferent connectivity onto oculomotor targets. The AF7-pretectal neurons innervate m-Rh5/6 and type Y motoneurons, compatible with commanding convergent saccades. By contrast, AF5-pretectal neurons have a connectivity pattern compatible with generation of conjugate eye movements.

### A model for saccade-type-specific recruitment of MRMNs

Our findings support a model in which two types of adducting saccades, with distinct kinematics and ethological roles, are controlled by parallel premotor pathways to produce saccade-type-specific recruitment of MRMNs (Figure 7E). We propose that GS MRMNs, located in dorso-medial nIII, are active for both conjugate and convergent adducting saccades. These motoneurons are recruited by contralateral INNs, which in turn are innervated by neurons in dorsal m-Rh5/6, forming a “saccade-type-agnostic pathway.” For convergent adducting saccades, a parallel “convergent saccade-specific pathway” leads to additional recruitment of type Y MRMNs in dorso-lateral nIII. Our data suggest that type Y motoneuron activity is influenced by a combination of afferent inputs including from INNs, ipsilateral ventral m-Rh5/6, and pretectal (APN) command neurons.

## Discussion

Our findings suggest that selective activation of subsets of extraocular motoneurons generates two types of kinematically distinct saccadic eye movement, and we identify parallel premotor pathways that are likely to control these rapid eye movements in a behavioral context-specific manner.

### Saccade-type-specific recruitment of extraocular motoneurons

To move the same body part with distinct kinematics, the nervous system must generate distinct patterns of ensemble motor unit activity. In the nIII, there has been a long-standing debate about the extent to which extraocular motoneurons might selectively participate in certain types of eye movement.^47^ In support of a final common pathway, recordings have shown that individual motoneurons participate in multiple types of eye movement (including saccades, slow vergence movements, and fixations).^9,31,48–51^ However, other studies have identified functional subtypes of motoneurons,^13^ and in primates, MRMNs show different position sensitivities during conjugate versus slow vergence eye movements,^11,12,52^ suggesting individual cells play a more or less important role during specific movement types. The concept of a final common pathway also appears at odds with conspicuous heterogeneity in the structure, biochemistry, and physiology of extraocular muscle fibers and their associated motoneurons. Specifically, extraocular muscles contain both fast-twitch singly innervated fibers (SIFs) and non-twitch multiply innervated fibers (MIFs), which are innervated by SIF and MIF motoneurons, respectively, with distinct neurochemistry, locations within the motor nuclei, and patterns of afferent innervation (reviewed in Horn and Straka^10^).

In this study, we used cellular-resolution calcium imaging during naturalistic behavior to provide direct evidence that subsets of extraocular motoneurons are selectively engaged during two types of saccadic eye movement. Their locations in the dorsal subdivision of nIII, activity during adduction of the ipsilateral eye, and positional overlap with neurons we reconstructed from EM data that receive monosynaptic INN input, collectively provide strong evidence that these cells are MRMNs.^31,32,35,53,54^ Our data support the notion that motoneuron recruitment is explicitly influenced by saccade type, independent of kinematic differences between the saccades we sampled. Specifically, the topography of MRMN activity, wherein dorso-medial MRMNs were active for both conjugate and convergent saccades, whereas dorso-lateral MRMNs are specifically recruited during convergent saccades, was consistent across a range of saccade amplitudes (and peak velocities, due to the main sequence relationship). Moreover, by comparing activity for kinematically matched pairs of saccades, we showed at the single-neuron level that motoneurons are explicitly modulated as a function of saccade type.

How might the parallel pathways function together to produce convergent saccades? Because convergent saccades of all sizes involve additional recruitment of type Y MRMNs, it might seem necessary that during small saccades there would be reduced pulse activity in the type-agnostic pathway, to account for their reduced velocity, as compared with conjugate saccades of equivalent amplitude. This is certainly plausible. While we named the pathway type-agnostic, the signals carried by premotor and motor neurons might nonetheless vary across saccade types (thus representing a hybrid of the two models in Figure 1A). Indeed, we observed negative saccade-type indices for some cells in dorso-medial nIII, nVI, and dorsal m-Rh5/6, indicating relatively greater activity during conjugate saccades. Intriguingly, a study in primates has also reported reduced MRMN population activity during disconjugate saccades (which contain a fast vergence component), as compared with pure conjugate saccades, leading to speculation that the “missing drive” might result from undersampling of smaller motoneurons.^54^ Our data suggest this could represent modulation of the primate equivalent of the type-agnostic pathway and that the missing population could be the primate equivalent of zebrafish type Y MRMNs.

### A synaptic mechanism for differential recruitment of MRMNs

Ultrastructural data indicated that differences in synaptic connectivity likely contribute to saccade-type-specific recruitment of extraocular motoneurons. We discovered three subtypes of MRMNs, with distinct dendritic morphologies and patterns of connectivity with INNs and which occupied different positions in dorsal nIII, aligned with the functional topography revealed by calcium imaging. Most surprising was the discovery of the GS motoneurons, which were located in dorso-medial nIII and whose somata enveloped a single, enormous presynaptic bouton, in a one-to-one connectivity pattern with a presynaptic INN. To our knowledge, this remarkable synapse has not previously been described in any species. The massive presynaptic terminal, containing abundant vesicles, mitochondria, and multiple active zones, is reminiscent of the calyx of Held in the auditory brainstem^55^ and the spoon endings of the avian tangential nucleus.^56^ It seems likely that it provides a fast and reliable feed-forward relay of INN activity, compatible with the fact that INNs show similar activity to extraocular motoneurons (including pulse-step signals during saccades^57^), as well as the similarity in functional PC1 scores we observed between dorso-medial MRMNs and INNs. Type X and type Y motoneurons also received synaptic input from INNs, but they had larger and more complex dendritic arbors, suggesting their recruitment is influenced by other afferent pathways. Indeed, for type Y motoneurons, we observed several additional sources of innervation, including from ventral m-Rh5/6 and pretectum. Based on these results, we propose that specific patterns of afferent innervation underlie saccade-type-specific recruitment of MRMNs.

In primates, tracing studies have identified three pools of MRMNs in and around nIII,^58^ which differ in size, afferent innervation, and synapse organization, leading to the suggestion that they might play distinct oculomotor roles.^37,59^ MIF motoneurons reside specifically in the “C-group,” and it has been suggested that they are specialized for slow vergence eye movements.^60^ Due to the limited size of the EM volume,^40^ we were unable to fully reconstruct MRMN axons and their terminations on muscle fibers, and so we could not identify the *en grappe* or *en plaque* synapses that are characteristic of MIF and SIF innervation, respectively.^61^ Future studies will establish which of the three subtypes of zebrafish MRMNs correspond to MIF and SIF motoneurons and how saccade-type-specific neural activity and kinematics relate to differences in activation of distinct muscle fiber types. Furthermore, what role type X motoneurons play is not yet clear and must await further tracing of their afferent inputs as well as MRMN-type-specific activity recordings. In any case, our data suggest that the subdivision of MRMNs into three subtypes with specialized functions might be widely conserved across modern vertebrates, from fish to primates.

### Premotor control of adducting saccades in different behavioral contexts

In our model, both types of adducting saccades depend upon GS MRMNs being recruited by contralateral INNs. INNs have been long understood to mediate conjugate eye movements by coupling activity in the nVI to contralateral the nIII to produce coordinated rotation of both eyes.^35,57^ While INNs are not required for slow vergence movements in mammals,^62–64^ we show that they are essential for fast convergent saccades in zebrafish. This necessitates that activity of INNs be uncoupled from LRMNs, and indeed, our calcium imaging revealed INN recruitment during both saccade types but minimal activity in LRMNs during convergent saccades when the ipsilateral eye must rotate nasally. This independence of the two neuronal types in nVI is supported by a recent connectomics study, which identified two oculomotor submodules in the zebrafish tegmentum, which are preferentially connected to either INNs or LRMNs.^65^ Furthermore, studies in mammals and fish have described monocular encoding in various oculomotor cell types, including the excitatory burst neurons (EBNs) that encode saccade velocity.^28,54,66–70^ In zebrafish, a likely candidate for these EBNs are the neurons we reconstructed in dorsal m-Rh5/6. Optogenetic mapping in larval zebrafish has previously identified rhombomere 5 as a locus capable of eliciting horizontal saccades,^27,41^ and like EBNs in mammals,^71–73^ dorsal m-Rh5/6 neurons are located in the PRF medial to nVI and make monosynaptic connections onto INNs and LRMNs. In line with our model, precise multiphoton optogenetic stimulation of small groups of cells within m-Rh5/6 reliably evoked adduction of the contralateral eye. Moreover, different stimulation sites showed substantial variability in their effects on the ipsilateral versus contralateral eye, in line with monocular encoding in EBNs.^66^ Although optogenetically evoked eye movements were slower than saccades, electrical stimulation of the PRF in mammals also tends to produce rather slow, constant velocity eye rotations, which has been interpreted to be a consequence of peripheral oculomotor circuits mediating a high degree of activity integration.^74,75^ Indeed, strong interconnectivity of this region with the horizontal velocity-to-position neural integrator (hVPNI) is suggested by both physiological and connectomics data in zebrafish, which indicate that highly recurrent hVPNI circuits extend through the tegmentum from Rh7-8 as far rostrally as Rh4-6.^65,76–80^

Zebrafish generate conjugate saccades in several contexts, including to visually scan their environment when stationary, shift or maintain gaze during locomotion, and to recenter the eye during OKR.^17^ All conjugate saccades conform to the same main sequence relationship, and so it is likely that saccadic commands from several brain regions converge upon dorsal m-Rh5/6 EBNs. Here, we identified AF5-pretectal projection neurons, which synapsed onto m-Rh5/6 neurons as well as LRMNs and INNs. It is unclear if these cells command saccades or other types of conjugate eye movement. Given that optogenetic stimulation of this region of pretectum (and electrical stimulation of its supposed mammalian equivalent) evokes slow phase eye movements,^43,81^ it is possible that AF5-pretectal neurons might mediate the early direct component of the OKR in which the eye responds to the onset of visual motion with a rapid increase in slow phase velocity.^82,83^ A recent study discovered a population of hindbrain neurons that display pre-saccadic ramping activity, which predicts the occurrence of spontaneous saccades.^29^ In future work, it will interesting to determine if and how these cells, as well as other afferent neurons, interface with the saccade-type-agnostic pathway we have proposed.

Convergent saccades are used by zebrafish to binocularly foveate their prey and to switch into a predatory mode of gaze during hunting.^17,18^ We propose that this saccade type is generated by additional activation of a parallel premotor pathway that recruits type Y MRMNs. Previously, we showed that neurons in the pretectal APN function as a hunting command system,^30^ and here, we show that they directly synapse onto type Y MRMNs, providing a link between induction of hunting state in the forebrain and a behavioral-state-specific oculomotor output. Stimulation of APN cells is sufficient to induce convergent saccades, and their connectivity to m-Rh5/6 suggests they might function as long-lead burst neurons, providing phasic activity that recruits EBNs. Type Y motoneurons also received an ipsilateral input from neurons in ventral m-Rh5/6, which were tuned to adduction of the ipsilateral eye and showed convergence-specific activity. At this stage, we can only speculate as to their function. In mammals, ipsilateral ascending inputs to MRMNs derive from ascending tract of Dieters (ATD) neurons in lateral and medial vestibular nucleus (LV, MV)^34,84^ and the nucleus prepositus hypoglossi (NPH),^85^ which forms part of the hVPNI. The cells we described might plausibly be part of MV (D. Schoppik, personal communication; Schoppik et al.^86^), yet recordings of ATD neurons in cats suggest they primarily convey a head velocity signal, albeit with some unreliable saccade-related activity.^87–89^ Moreover, NPH neurons innervating nIII have an ON-direction corresponding to abduction of the ipsilateral eye, opposite to our results. Therefore, it is possible that the cells we have identified represent a previously undescribed input to MRMNs. Their location in m-Rh5/6 suggests they may function as burst neurons, providing type Y cells with an eye velocity signal, while the putative relationship with NPH raises the possibility that they (perhaps additionally) carry an eye position signal. Zebrafish sustain high ocular vergence for the duration of hunting sequences,^18^ necessitating a tonic signal to maintain eye position after saccadic convergence, which is presumably generated by a neural integrator. Although we have not explored the matter in depth, our preliminary analysis suggests that eye position decays with a longer time constant following convergent saccades, and lesioning of INN axons in the medial longitudinal fasciculus (MLF) causes a significant deficit in gaze holding only following conjugate adducting saccades (data not shown). This raises the possibility that there might be a separate vergence integrator (i.e., in addition to the hVPNI) and that the eye position signal it generates might reach type Y neurons via the MLF-independent pathway from ventral m-Rh5/6. We note that sustained activity in APN neurons could also contribute to maintaining or adjusting post-saccadic vergence. Future work will clarify the signals carried by these premotor elements and resolve how they cooperate with INN innervation to recruit type Y MRMNs and sustain eye position during predatory eye convergence.

### Brainstem control of fast vergence

How does our model compare with what is understood of fast vergence control in primates? The convergent saccades of larval zebrafish^17^ are similar to disjunctive saccades (DSs) of primates,^90^ which also comprise version (conjugate) and vergence components to shift fixation between targets in 3D space. Although these are by far the most common saccades used in everyday viewing, their neural basis has received little attention, as compared with conjugate saccades and slow, symmetric vergence (driven by accommodative and disparity signals).^91^ Previous models have sought to explain DSs by combining activity of the (conjugate) saccade and vergence subsystems in a schema that accords with Hering’s ideas about binocular coordination of eye movements, in which identical commands are sent to both eyes.^92^ To account for high vergence velocities during DSs, such models hypothesized the existence of an additional neural component, saccade-vergence burst neurons (SVBNs), and recently, cells carrying the appropriate signal were discovered in the central mesen-cephalic reticular formation of monkeys.^93^ We were not able to identify cells with activity similar to SVBNs, nor cells encoding vergence position or velocity,^94–97^ suggesting zebrafish are unlikely to have a midbrain vergence subsystem. However, recent work has challenged the idea that such a system is required for DSs, perhaps even in primates. Specifically, the finding that a wide variety of neurons in the “conjugate” saccadic pathway appear to carry monocular signals supports a model in which each eye is programed independently, and therefore the saccadic system can mediate both the version and vergence components of DSs.^91^ Our data support and extend this model by proposing that both the canonical saccadic pathway and an additional parallel pathway act together to generate fast vergence eye movements. The circuits we have mapped in larval zebrafish might represent an ancestral vertebrate blueprint for the control of fast vergence, and later in evolution, the appearance of a midbrain vergence system may have arisen for more precise binocular foveation and stereopsis.^64^ Finally, although we define convergent and conjugate saccades in a binocular context, here we have outlined pathways that control adduction of a single eye. In future work, we hope to leverage the experimental accessibility of the larval zebrafish brain to understand how the animal coordinates both eyes to binocularly foveate its prey.

## Resource Availability

### Lead contact

Further information and requests for resources and reagents should be directed to and will be fulfilled by the lead contact, Isaac H. Bianco (i.bianco@ucl.ac.uk).

### Materials availability

Constructs and transgenic lines generated in this study are available from the lead contact on request.

## Star★Methods

Detailed methods are provided in the online version of this paper and include the following:

- KEY RESOURCES TABLE
- EXPERIMENTAL MODEL AND STUDY PARTICIPANT DETAILS
  ○ Zebrafish lines and care
  ○ Generation of transgenic zebrafish
- METHOD DETAILS
  ○ Two-photon calcium imaging and behavioural tracking
  ○ Saccade detection and classification
  ○ Calcium imaging analysis
  ○ Photoactivation of paGFP
  ○ Analysis of electron microscopy data
  ○ Transmission electron microscopy
  ○ Image registration
  ○ Laser ablations
  ○ 2-photon optogenetics
- QUANTIFICATION AND STATISTICAL ANALYSIS

## Star★Methods

### Key Resources Table

**Table:** 

REAGENT or RESOURCE	SOURCE	IDENTIFIER
Deposited data		
Processed imaging and behavioural data	–	Mendeley Data, v1: https://doi.org/10.17632/6dtxb5zvnj.1
Experimental models: Organisms/strains		
Tg(elavl3:H2B-GCaMP6s)jf5Tg	Freeman et al.^98^	ZFIN: ZDB-ALT-141023-2
Tg(elavl3:jRCaMP1a)jf16Tg	Dunn et al.^99^	ZFIN: ZDB-ALT-160519-3
Tg(Cau.Tuba1:c3paGFP)a7437Tg	Bianco et al.^100^	ZFIN: ZDB-ALT-120919-1
Tg(UAS:CoChR-tdTomato)u332Tg	Antinucci et al.^101^	ZFIN: ZDB-ALT-190226-4
Tg(pvalb6:KalTA4)u523Tg	This paper	N/A
Software and algorithms		
MATLAB 2021a	Mathworks	https://uk.mathworks.com/products/new_products/release2021a.html
UMAP	Meehan et al.^102^	https://uk.mathworks.com/matlabcentral/fileexchange/71902-uniform-manifold-approximation-and-projection-umap
Saccade classification	Dowell et al.^17^	https://data.mendeley.com/datasets/vd5zdfwc37/1
Advanced Normalization Tools (ANTs)	Avants et al.^103^	https://stnava.github.io/ANTs/
LabVIEW	National Instruments	https://www.ni.com/en/shop/labview.html
Psychophysics Toolbox	Brainard^104^	http://psychtoolbox.org/
Cell detection	Kawashima et al.^105^	https://github.com/ahrens-lab/Kawashima_et_al_Cell_2016/
Knossos	–	https://github.com/knossos-project/knossos
Blender 3.4	Blender	https://www.blender.org/download/releases/3-4/
Code to plot figures	This paper	Mendeley Data, v1: https://doi.org/10.17632/6dtxb5zvnj.1

### Experimental Model and Study Participant Details

#### Zebrafish lines and care

Zebrafish lines were maintained in the Tübingen background. Larvae were reared in fish-facility water on a 14/10 h light/dark cycle at 28:5° C and were fed *Paramecia* from 4 dpf onwards. All larvae were homozygous for the *mitfa w*2^106^ skin-pigmentation mutation. For functional imaging, animals were transgenic for Tg(elavl3:H2B-GCaMP6s)jf5Tg.^98^ Photoactivations were performed using larvae carrying Tg(Cau.Tuba1:c3paGFP)a7437Tg^100^ and Tg(elavl3:jRCaMP1a)jf16Tg.^99^ Optogenetic stimulation was conducted using Tg(KalTA4u523);Tg(UAS:CoChR-tdTomato)u332Tg (below and^101^). The sex of the larvae is not defined at the early stages of development used for these studies. Experimental procedures were approved by the UCL Animal Welfare Ethical Review Body and the UK Home Office under the Animals (Scientific Procedures) Act 1986.

#### Generation of transgenic zebrafish

The Tg(–2.5pvalb6:KalTA4)u523Tg [abbreviated Tg(KalTA4u523)] transgenic line was isolated by screening the progeny of animals injected with a –2.5*pvalb6*:KalTA4 expression construct that was generated and injected as described in Antinucci et al.^30^ This expression vector generated a wide range of expression patterns, one of which was designated the allele u523Tg and labelled a broad population of neurons in midbrain and hindbrain.

### Method Details

#### Two-photon calcium imaging and behavioural tracking

Larvae were tethered in 3% low-melting point agarose gel in a 35 mm petri dish lid and sections of gel were carefully removed using an opthalmic scalpel to allow free movement of the eyes and tail below the swim bladder. Larvae were allowed to recover overnight before testing at 6 or 7 dpf. Imaging was performed using a custom-built multiphoton microscope [Olympus XLUMPLFLN × 20 1.0 NA objective, 580 nm PMT dichroic, bandpass filters: 510/84 (green), 641/75 (red) (Semrock), R10699 PMT (Hamamatsu), Chameleon II ultrafast laser (Coherent)] at 920 nm with laser power at sample of 5–10 mW. Images (0.67 *μ*m/px) were acquired by frame scanning at 4.8 Hz, with focal planes separated by 10*μ* m.

Eye position was monitored at either 60 or 300 Hz using a FL3-U3-13Y3M-C camera (Point Grey) that imaged through the microscope objective under 720 nm illumination. Tail position was imaged at 420 Hz under 850 nm illumination using a sub-stage GS3-U3-41C6NIR-C camera (Point Grey). Horizontal eye position and tail posture (defined by 13 equidistant x-y coordinates along the anterior-posterior axis) were extracted online using machine vision algorithms.^107^

Two projectors were used to present visual stimuli. The first (Optoma ML750ST) back-projected stimuli onto a curved screen placed in front of the animal at a viewing distance of 35 mm while the second (AAXA P2 Jr) projected images onto a diffusive screen directly beneath the chamber. Visual stimuli were defined using the ‘red’ colour channel and Wratten filters (Kodak, no. 29) were placed in front of both projectors to block residual light that might be detected by the PMT. Visual stimuli were designed in MATLAB using Psychophysics Toolbox.^104^ Prey-like moving spots comprised 5∘ bright or dark spots (Weber contrast +1 or -1 respectively) moving at 30∘/s either left → right or right → left across 152° of frontal visual space. Optokinetic stimuli were presented in front of the animal and comprised drifting sinusoidal gratings (wavelength 19°, velocity 0.3 cycles/s, Michelson contrast 0.5) that alternated between leftwards or rightwards motion every few seconds. Stimuli were presented in a pseudo-random sequence with a 30 s inter-stimulus interval.

Microscope control, stimulus presentation and behaviour tracking were implemented using LabView (National Instruments) and MATLAB (MathWorks).

#### Saccade detection and classification

Saccadic eye movements were analysed as per Dowell et al.^17^ Raw eye position traces were first interpolated onto a 100 Hz time-base and low-pass filtered with a cut-off frequency of 1 Hz. Rapid eye movement events were detected as peaks in the convolution of filtered eye position with a step function (width 160 ms), with the time of the peak providing a first coarse estimate of saccade time. Rapid eye movement events of the left and right eye that occurred within 100 ms of one another were paired and treated as a single binocular event. After this pairing step, events that occurred within 300 ms of a preceding event were discarded, to limit overlap between windows for calculating saccade metrics (see below) and because manual inspection revealed these movements were rarely saccadic.

To reliably estimate eye position and velocity metrics, raw eye position traces were interpolated onto a 500 Hz timebase and smoothed with a custom LOWESS function, which was designed to reduce noise without ‘flattening’ changes in eye position during saccades. Specifically, eye position data was smoothed using the MATLAB lowess function (with span 80 ms) except for periods where the convolution of raw eye position data with a step function exceeded 3° (putative saccades). A refined estimate of saccade onset time was then determined by convolving smoothed eye position with two step functions of width 100 ms and 40 ms, taking the product between both convolutions and thresholding the output within a 400 ms window spanning the initial estimate of saccade time.

For each rapid eye movement event we evaluated: (a) pre-saccadic eye position, as median eye position during a 200 ms window immediately prior to onset time; (b) max post-saccadic eye position, as the eye position within a 200 ms window starting at onset time that had the greatest absolute deviation from eye position at onset time; (c) median post-saccadic eye position, as median eye position over a 200 ms window starting at the timepoint corresponding to max post-saccadic eye position; (d) eye velocity (cw and ccw), as the maxima and minima, respectively, of the time derivative of eye position, determined by the MATLAB gradient function over a 150 ms window centred at onset time. We then used these measures to calculate nine oculomotor metrics describing each (binocular) rapid eye movement event: *Amplitude* (left and right eye), was the difference between median post-saccadic eye position and pre-saccadic eye position. *Max-median amplitude* (left and right eye), was the difference between max post-saccadic eye position and median post-saccadic eye position and quantifies the degree to which eye position is maintained following a saccade. *Velocity* (cw and ccw for both left and right eye), as described above. *Vergence*, was the difference between median post-saccadic eye position of the right and left eye. These metrics were normalized for each animal by winsorizing the data between the 0.5th and 99.5th percentile and then z-scoring.

To classify rapid eye movement events and label specific saccade types, we first used a MATLAB implementation^102^ of UMAP^108^ (run_umap, metric=Euclidean, min_dist=0.11, n_neighbours=199) to perform a supervised embedding into a two-dimensional UMAP space previously derived from 152 tethered animals.^17^ Then, for each rapid eye movement event, a saccade-type identify was chosen by taking the modal identity of 100 nearest neighbours in the embedding space. In this study, we restricted our analysis to left conjugate, right conjugate and convergent saccades. For regression modelling of calcium time series (below), left and right lateralised convergent saccades were distinguished by the sign of post-saccadic version (the average of left and right eye position).

#### Calcium imaging analysis

##### Image processing and time-series extraction

Motion correction of fluorescence imaging data was performed as per.^107^ Regions of interest (ROIs), corresponding to GCaMP labelled nuclei of individual neurons, were segmented using an algorithm from.^105^ The fluorescence time series of each cell was initially computed as the mean value of pixels belonging to the corresponding binary mask for each imaging frame, assigned to a time point corresponding the midpoint of the frame. For frames where motion error exceeded 5 *μ*m, pixel values were replaced by interpolation. This initial time series estimate was then detrended to correct for slow variations in fluorescence and standardised by (1) subtracting baseline fluorescence, estimated as the 50th percentile of pixel values and (2) dividing by the ‘noise’ of the calcium signal baseline, estimated using the OASIS^109^ subfunction estimate_baseline_noise. The resulting fluorescence time-series is denoted *zF*.

##### Oculomotor-tuned ROIs

Oculomotor-tuned ROIs were identified using a two-stage analysis. First, we identified ROIs as being ‘saccade-active’. Second, we identified ‘oculomotor-tuned’ ROIs as the subset of saccade-active ROIs whose fluorescence was best explained by oculomotor variables, as opposed to other stimulus or motor variables.

To identify saccade-active ROIs, we first computed for each ROI three d’ values, one for each saccade type (i.e. convergent saccades (*Conv*) and left and right conjugate saccades (*L Conj, R Conj*)), where

$$d^{\prime }=\frac{\mu_{post}-\mu_{pre}}{\sqrt{\frac{\sigma_{post}^{2}+\sigma_{pre}^{2}}{2}}}$$

and *μ*_*post*_ and $\sigma_{post}^{2}$ were the mean and variance of zF across time and individual instances of the saccade type during a 2 s window following saccade onset. *μ*_*pre*_ and $\sigma_{pre}^{2}$ were computed similarly during a 1 s window prior to saccade onset. For each ROI and saccade type, a null distribution of 1000 d’ values was generated by randomising saccade onset times. An ROI was considered active for a given saccade type if the relevant d’ value exceeded the 95th percentile of this null distribution.

In the second step, we used linear regression to model *zF* for each saccade-active ROI. We designed 33 regressors, derived from 14 behavioural predictors and 18 stimulus predictors (Table S1). Six ‘oculomotor predictors’ comprised four predictors describing the occurrence of saccades and two eye position predictors. The saccadic predictors were one-hot encodings indicating the imaging frames corresponding to saccade onset for leftward- and rightward-directed convergent and conjugate saccades. In view of the fact that many oculomotor neurons encode ipsi- or contraversive eye position, we generated rectified eye position predictors for the left and right eye, where temporal eye positions (estimated as those positions more temporal than the median position across the entire experiment) were zeroed. Locomotor predictors were one-hot encodings of the onset time of swims, specific for direction (left/right) and vigour level (1st to 4th quartile). Of the 18 stimulus predictors, two described optokinetic drifting gratings and the remainder described small moving spots. The optokinetic grating predictors were binary vectors indicating the frames during which left- or right-wards motion was presented. Small spot predictors were one-hot encodings describing a range of stimulus locations (from -60 to +60 degrees azimuth), specific for motion direction and contrast polarity. To account for calcium dynamics, regressors were generated by convolving each predictor with a calcium impulse response function (CIRF) modelled as an exponential rise and subsequent decay with time constants *τ*_*on*_ = 0.2 s and *τ*_*off*_ = 3–5 s (see below). In addition, to capture fluorescence modulations that might result from residual motion artefacts, we included a ‘motion-error’ regressor, derived from the translation applied during motion correction of image time series (this regressor was not convolved with the CIRF).

First, for each saccade-active ROI, we used ordinary least squares regression to optimise two hyperparameters: The τ_*off*_ of the CIRF (3, 4 or 5 s) and a temporal offset applied to the regressors (0, 1, 2 or 3 frames). ROIs for which the best OLS model had *R*^2^ > 0.05 (67.8±9.3% of saccade-active ROIs, *N* = 76 fish) were then subjected to (more computationally intensive) ridge regression using the best performing pair of hyperparameters.

We used regularized linear regression with an L2 penalty (‘ridge’ regression) to model zF for selected ROIs. Ridge regression was performed using the MATLAB ridge function, with lambda selected by ten-fold cross-validation. To estimate the unique contribution of each regressor to the model fit, we followed the approach of.^110^ Specifically, for each regressor in turn, we circularly permuted the regressor by a random number of frames and recomputed the model fit using the same lambda value and cross-validation folds as per the original fit. In this way we derived a change in cross-validated goodness-of-fit (Δ*cvR*^2^) resulting from randomising the temporal relationship between the regressor in question and the recorded calcium fluorescence of the ROI. Negative values of Δ*cvR*^2^ indicate the regressor made a unique contribution to explaining ROI activity that could not be compensated by the remaining regressors. Positive values of Δ*cvR*^2^ represent random improvements in fit quality from permuting the predictor; we pooled these values across ROIs to generate a null-distribution and estimate significant values of Δ*cvR*^2^. Thus, a saccade-active ROI was classified as oculomotor-tuned if (i) the most negative Δ*cvR*^2^ value was associated with an oculomotor regressor; (ii) at least one oculomotor regressor had a Δ*cvR*^2^ more negative than the 95th percentile of the null distribution; (iii) the motion artefact predictor was *less* negative than the 95th percentile of the null. In this way we identified oculomotor-tuned ROIs whose zF time-series was best predicted by an oculomotor variable and was not explained by residual motion error. These ROIs were labelled as being active for ‘Conv’, ‘L Conj’ or ‘R Conj’ saccades, or ‘Both’ if the d’ analysis indicated significant activation for convergent as well as either left or right conjugate saccades (see Venn diagram in Figure S1F).

##### Oculomotor tuning metrics

Rectilinear fits and saccade type index were determined from normalised saccade-triggered fluorescence and normalised post-saccadic eye position and eye velocity. For each ROI, normalised saccade-triggered fluorescence was calculated by first subtracting from *zF* its mean value over a 1 s window immediately prior to saccade onset and then summing *zF* over a 2 s window starting at saccade onset, thus providing a measure of saccade-triggered fluorescence change. These values were then divided by their 95th percentile across all saccades for a given ROI to provide a set of normalised saccade-triggered fluorescence values.

Post-saccadic eye position was normalised by dividing by the maximum post-saccadic nasal eye position across all saccades. Eye velocity was normalised by dividing by the 95th percentile of nasal eye velocities across saccades.

Rectilinear fits of normalised saccade-triggered fluorescence versus normalised post-saccadic eye position consisted of a horizontal baseline, equal to median fluorescence across a given span and a linear ramp, which was computed by least-squares regression starting at a threshold post-saccadic eye position. Successive fits were computed with baselines spanning progressively larger portions of the data and the fit with the lowest mean-squared error was selected if the ramping portion increased above/below threshold. Otherwise only a baseline was fit. Separate fits were made for abducting and adducting saccades for each eye.

Saccade type index was estimated by first matching conjugate saccades with kinematically similar convergent saccades for a given eye. Specifically, for each conjugate adducting saccade, a matched convergent adducting saccade was found when its Euclidean distance was < 0.1 in normalised post-saccadic eye position and velocity space. If more than one convergent saccade fell within this radius, the closest was selected. Next, for each ROI, we computed the median difference between normalised saccade-triggered fluorescence across these matched saccade pairs. For a proportion of ROIs (18%), saccade type index could not be computed because there were no matched pairs of saccades.

For computing saccade type indices, the activity of each ROI had to be considered with respect to either the left or right eye. To select the appropriate eye, a directionality preference was established by summing Δ*cvR*^2^ values corresponding to leftwards (LConj, ConvGL, and right eye nasal position) or rightwards (RConj, ConvGR, and left eye nasal position) eye movements; the more negative sum specified directionality preference. ROIs with a preference for leftward movement had saccade type index and nasal ramp fits computed with respect to the right eye and temporal ramp fits computed with respect to the left eye. For ROIs with rightwards preference, the eyes were reversed. In support of this approach, the best ramp fit corresponded to this directionality preference for the vast majority (83%) of ROIs.

The OKR power metric was designed to quantify activity modulation associated with slow phase eye movements during the optokinetic response. When averaged over multiple OKR presentations, eye position traces showed periodic movement that followed the direction of whole-field motion with the effects of reset saccades largely smoothed out. Thus, for each ROI, we computed the median of *zF* across presentations of leftwards and rightwards OKR gratings. Because these slow phase movements were offset by ~ 180°, we computed the difference between these median responses to isolate direction-selective phasic signals. We then computed the Fourier transform and assessed power spectral density at the frequency of OKR direction modulation to yield an OKR power score.

#### Photoactivation of paGFP

Larvae homozygous for Tg(Cau.Tuba1:c3paGFP)a7437Tg^100^ and Tg(elavl3:jRCaMP1a)jf16Tg^99^ were mounted in 1% low-melting point agarose at 5 dpf. The same custom 2-photon microscope used for functional calcium imaging was used for photoactivations. paGFP was photoactivated by continuously scanning a small volume at 790 nm, 5-10 mW power at sample for 3–8 min per plane. Photoactivation volumes were 40×40×20 – 30 *μ*m x-y-z with focal planes spaced 5 *μ*m apart. Animals were then unmounted and allowed to recover for 24 h after which paGFP and jRCaMP1a were imaged at 1040 nm.

Locations of retrogradely labelled somata were manually determined from image volumes registered to ZBB coordinate space. The axon terminals of abducens internuclear neurons were measured using ImageJ from high-resolution stacks (0.1×0.1×1 – 2 *μ*m/px) in oculomotor nucleus. The sizes of axonal boutons were measured in the focal planes within which they had the largest cross-sectional area.

#### Analysis of electron microscopy data

For ultrastructural reconstruction of neurons we used a publicly available serial-blockface electron micrograph volume from a 5 dpf larval zebrafish acquired at 14×14×25 nm.^40^ The dataset consisted of automatically detected and over-segmented cell bodies and neurites, which we manually agglomerated into neuron morphologies using the Knossos open source software (https://github.com/knossos-project/knossos). Since our aim was to identify connections between functionally defined brain regions, we did not necessarily fully reconstruct every neuron; in any case, numerous artefacts in the dataset often precluded this.

Reconstructions of abducens internuclear neurons began either from cell bodies in the abducens nucleus, or from large axon terminals in the oculomotor nucleus. Complete axon morphologies and partial dendritic morphologies were reconstructed by merging segments in ‘Agglomeration’ mode in Knossos. Segments that terminated within a neurite and thus over-segmented the neuron, were merged. Neurites were visualised in 3 orthogonal views of the raw EM data and followed until they terminated, merged with the cell body or until we encountered an artefact in the data.

Extraocular motoneurons were reconstructed within the oculomotor nucleus (nIII) from sites post-synaptic to abducens internuclear neurons and are therefore assumed to be medial rectus motoneurons.^35^ Synapses were identified according to four criteria: (1) Close apposition between the two membrane surfaces; (2) identification of punctate dark spots close to the opposing membranes, consistent with pre-synaptic vesicles; (3) darkening or thickening of the post-synaptic membrane; (4) the membrane apposition had been classified as a synapse by the automated synapse detection algorithm deployed in.^40^ Reconstructed medial rectus motoneurons extended their axons into the third cranial nerve, as expected. Although we reconstructed the dendritic and axon morphologies as fully as possible, because the dataset did not encompass the extraocular muscles, it was not possible to identify post-synaptic targets or classify extraocular motoneurons as MIFs or SIFs.

Almost all neurons pre-synaptic to internuclear neurons and Type-Y motoneurons were traced from the pre-synaptic terminal (identified using the above criteria) back towards the cell body. One ventromedial rhombomere 5/6 neuron was traced from its cell body to Type Y motoneuron dendrites. Pretectal neurons were traced from the cell body as were the subset of ventral m-Rh5/6 neurons for which no connection to extraocular motoneurons was identified.

Morphologies were exported as .ply meshes and were plotted in Blender for visualisation (https://www.blender.org).

#### Transmission electron microscopy

Zebrafish larvae were fixed at 6 dpf by immersion for 24 h in EM fix [2% (w/v) paraformaldehyde, 2% (w/v) EM-grade gluteraldehyde, in 0.1 M sodium cacodylate buffer (pH 7.3); all fix reagents from Agar Scientific, Stansted, UK]. Specimens were then postfixed in 1% osmium tetroxide for 3 h, dehydrated in an ethanol series, infiltrated with medium hard AGAR100 resin (Agar Scientific, Stansted, UK) and polymerised by baking at 60° C for 24–48 h. Optimally orientated specimens were selected for sectioning from a dorsal approach. As sectioning proceeded, semi-thin (1 *μ*m thick) sections stained using Toluidine Blue were checked in batches of 5–10 until approximately the correct location was reached. This location was identified by comparing neuropil areas with the Svara et al., 2022^40^ EM dataset and ZBB light-microscopy dataset. Once the correct location was determined to have been reached, ultrathin (80 nm) sections were collected onto 2 mm Pioloform resin-coated copper slot grids (Agar Scientific, Stansted, UK). Grids were stained with uranium and lead stains and were examined using a JEOL JEM-1400Flash at 80 kV. Images were captured using a Gatan Rio16 digital camera.

#### Image registration

Registration of image volumes was performed using the ANTs toolbox version 2.1.0^103^ in a similar manner to that described in ^111^. Images were converted to NRRD file format for registration using ImageJ. As an example, to register the 3D image volume ‘fish1.nrrd’ to reference brain ‘ref.nrrd’, the following command was used:

antsRegistration -d 3 –float 1 -o [fish1, fish1_Warped.nii.gz] -n BSpline -r [ref.nrrd, fish1.nrrd,1] -t Rigid[0.1] -m C[ref.nrrd, fish1.nrrd,1,32, Regular,0.25] -c [200x200x200x0,1e-8,10] -f 12x8x4x2 -s 4x3x2x1 -t Affine[0.1] -m GC[ref.nrrd, fish1.nrrd,1,32, Regular,0.25] -c [200x200x200x0,1e-8,10] -f 12x8x4x2 -s 4x3x2x1 -t SyN[0.05,6,0.5] -m CC[ref.nrrd, fish1.nrrd,1,2] -c [200x200x 200x200x10,1e-7,10] -f 12x8x4x2x1 -s 4x3x2x1x0

The deformation matrices computed above were then applied to any other image channel N of fish1 using: antsApplyTransforms -d 3 -v 0 –float -n BSpline -i fish1-0N.nrrd -r ref.nrrd -o fish1-0N_Warped.nii.gz -t fish1_1Warp.nii.gz -t fish1_0GenericAffine.mat

All fluorescence imaging volumes were registered to the ZBB brain atlas^26^ and to a high-resolution reference brain [from one of the following transgenic lines: Tg(elavl3:H2B-GCaMP6s), Tg(elavl3:jRCaMP1a), Tg(elavl3:GCaMP7f) or Tg(u523:KalTA4);Tg(UAS: CoChR-tdTomato)], all with resolution 0.77×0.77×1 *μ*m/px. Registrations were conducted for different experiments as follows:

- For registration of calcium imaging data, a two-step registration process was used. First, functional calcium imaging volumes were registered to a volume of the same brain acquired at the end of the experiment with z-voxel dimension 1 *μ*m (‘post-stack’). Second, the post-stack was registered to the high-resolution Tg(elavl3:H2B-GCaMP6s) brain. Since the high-resolution brain was registered to ZBB atlas space, the transformations were concatenated to bring the functional imaging data and ROI locations to ZBB space (calcium imaging volume → post-stack → hi-res brain → ZBB).
- Image volumes of retrogradely photo-labelled somata were registered to high-resolution Tg(elavl3:jRCaMP1a) or Tg(elavl3:GCaMP7f) reference brains using Tg(elavl3:jRCamp1a) expression imaged in the red channel. Transformations were applied to the green channel to bring paGFP expression into ZBB space.
- Internuclear neuron axon terminals were registered using a two-step process. A small, high-resolution volume (0.1×0.1×1 − 2 *μ*m/px) encompassing the axon terminals was first registered to a larger volume (308×308×50 − 70 μm; 0.39×0.39×2 μm/px) which was in turn registered to the high-resolution Tg(elavl3:jRCaMP1a) reference brain.
- For each optogenetic stimulation site a post-stimulation image was acquired. This was manually aligned to an image volume of the whole brain acquired at the end of the experiment, which was in turn registered to the Tg(u523:KalTA4);Tg(UAS:CoChR-tdTomato) high-resolution reference brain.

#### Laser ablations

Somatic ablations were guided by anatomical location and calcium activity. Calcium imaging was performed using 6 dpf Tg(elavl3:H2B-GCaMP6s) animals and immediately after imaging maps of pixel-wise mean fluorescence modulation in response to whole-field motion, convergent and conjugate saccades were computed. Cells in the relevant anatomical region (nVI or m-Rh5/6) and which showed positive fluorescence modulations during convergent saccades, were identified. These target cells were ablated using the same custom 2-photon microscope described above, following the procedure of.^111^ In brief, animals were anaesthetised and the laser focus was spiral-scanned over the target soma for ~ 140 ms (800 nm, 150-200 mW at sample). Ablations were deemed successful if an auto-fluorescent ‘scar’ was subsequently visible in both green and red channels. Target locations in m-Rh5/6 were logged in 10 fish (out of 13) and for nVI in 4 fish (out of 7). Following the procedure, animals were unmounted and allowed to recover overnight before undergoing subsequent functional imaging and behavioural tracking.

Axotomies were performed to sever the axons of abducens internuclear neurons in the medial longitudinal fasciculus. The axons were visualised by photoconverting paGFP in nVI at 5 dpf (see above). At 6 dpf, pre-ablation behavioural data was collected and then axotomies were performed using a similar ablation procedure, except that we used higher laser power (250–290 mW). Multiple sites were often targeted to ensure all photolabelled axons were cut. Behaviour was assayed post-ablation following overnight recovery.

#### 2-photon optogenetics

Optogenetic photostimulation of CoChR-expressing neurons was performed using Tg(u523:KalTA4);Tg(UAS:CoChR-tdTomato) transgenic animals (6–7 dpf) which have widespread opsin expression in mid/hindbrain tegmentum. Stimulation was performed by raster-scanning small areas (6–20 × 6–20 *μ*m at 24–45 Hz, 920 nm, 17 mW) for 4–8 s with stimulation trials separated by 15–20 s intervals. Eye and tail movements were tracked throughout and no background illumination was provided. Changes in eye position were calculated as the difference between median eye position 250 ms prior to stimulus onset and 250 ms prior to stimulus offset. Optogenetic stimulation sites were mapped to ZBB coordinates.

### Quantification and Statistical Analysis

All statistical analyses were performed in MATLAB. Types of statistical test and *N* are reported in the text or figure legends. All tests were two-tailed and we report *p*-values without correction for multiple comparisons unless otherwise noted.

## Supplementary Material

**Supplemental Information**

Supplemental information can be found online at https://doi.org/10.1016/j.cub.2024.12.010.

Supplementary Material

## Data Availability

The code and pre-processed data to generate the figures in this manuscript are publicly available at Mendeley: https://data.mendeley.com/datasets/6dtxb5zvnj/1. The raw data generated in this study will be shared by the lead contact upon request, but it has not been deposited due to its large size.

## Highlights

- Subsets of extraocular motoneurons control two types of adducting saccades
- Recruitment of medial rectus motoneurons is controlled by distinct synaptic inputs
- Parallel premotor pathways recruit motoneuron subsets in a context-specific manner
- Forebrain hunting commands directly interface onto hunting-specialized motoneurons
