# Supplementary material for: Subsets of extraocular motoneurons produce kinematically distinct saccades during hunting and exploration
Source: Curr Biol. Author manuscript; Available in PMC 2025 Dec 20. (PMC7618498; doi:10.1016/j.cub.2024.12.010)
Supplement: Supplementary Material [file EMS211315-supplement-Supplementary_Material.pdf]

Current Biology, Volume *35*

Supplemental Information

Subsets of extraocular motoneurons produce kinematically distinct saccades during hunting and exploration

Charles K. Dowell, Thomas Hawkins, and Isaac H. Bianco

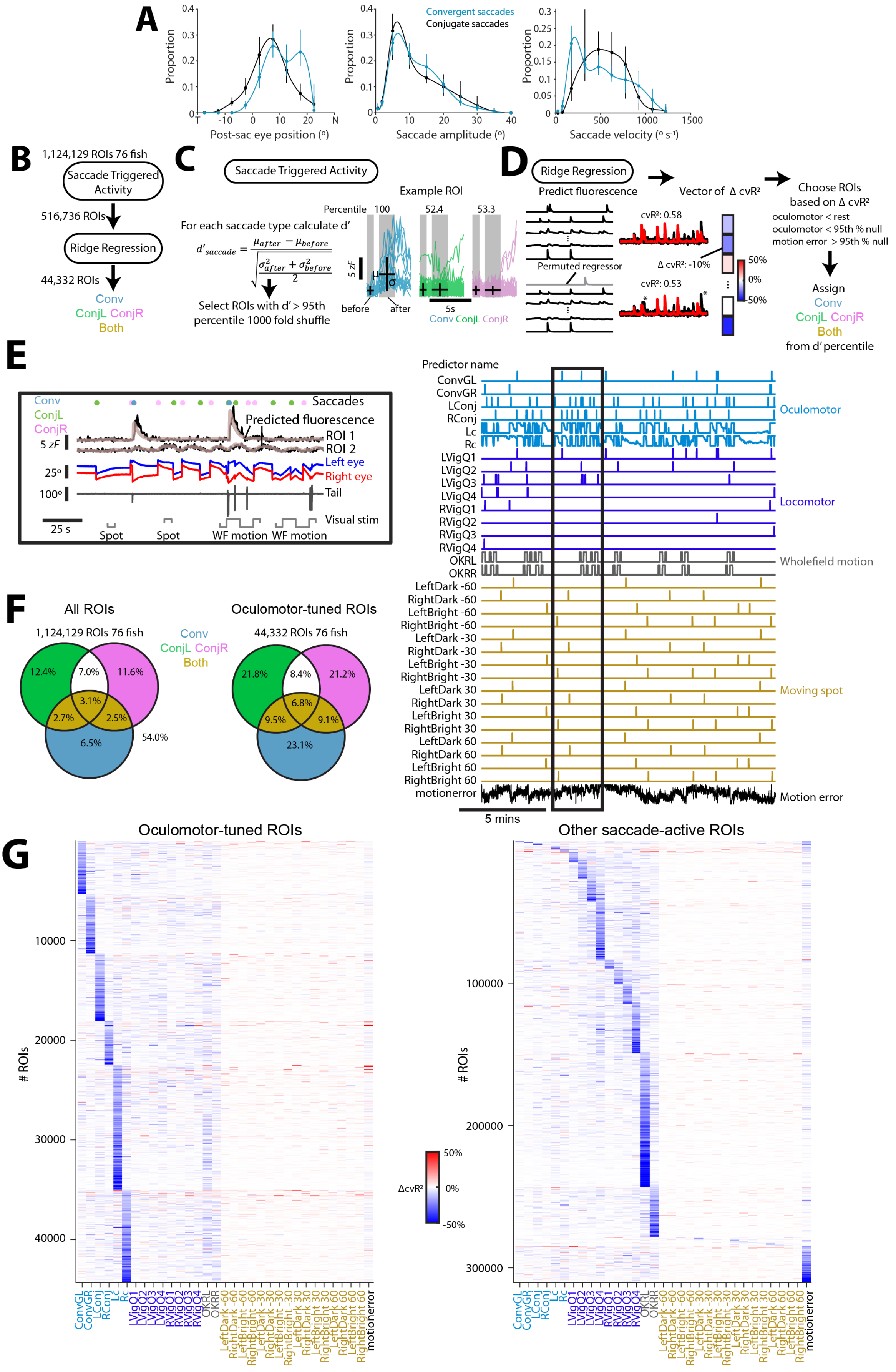
A
Proportion
Post-sac eye position (°)
Convergent saccades
Conjugate saccades
Saccade amplitude (°)
Saccade velocity (° s-1)
B
1,124,129 ROIs 76 fish
Saccade Triggered Activity
516,736 ROIs
Ridge Regression
44,332 ROIs
Conv
ConjL ConjR
Both
C
Saccade Triggered Activity
For each saccade type calculate d'
Select ROIs with d' > 95th percentile 1000 fold shuffle
Example ROI
Percentile 100 52.4 53.3
before
after
5s
Conv ConjL ConjR
D
Ridge Regression
Predict fluorescence
Permuted regressor
cvR²: 0.58
cvR²: 0.53
Δ cvR²: -10%
Vector of Δ cvR²
Choose ROIs based on Δ cvR²
oculomotor < rest
oculomotor < 95th % null
motion error > 95th % null
Assign
Conv
ConjL ConjR
Both
from d' percentile
E
Saccades
Predicted fluorescence
ROI 1
ROI 2
Left eye
Right eye
Tail
Visual stim
25 s
Spot
Spot
WF motion
WF motion
Predictor name
Oculomotor
Locomotor
Wholefield motion
Moving spot
Motion error
5 mins
F
All ROIs
1,124,129 ROIs 76 fish
Oculomotor-tuned ROIs
44,332 ROIs 76 fish
12.4%
7.0%
11.6%
3.1%
2.7%
2.5%
6.5%
54.0%
21.8%
8.4%
21.2%
6.8%
9.5%
9.1%
23.1%
G
Oculomotor-tuned ROIs
Other saccade-active ROIs
ROIs
ΔcvR²
50%
0%
-50%
ConvGL ConvGR LConj RConj Lc Rc LVigQ1 LVigQ2 LVigQ3 LVigQ4 RVigQ1 RVigQ2 RVigQ3 RVigQ4 OKRL OKRR LeftDark -60 RightDark -60 LeftBright -60 RightBright -60 LeftDark -30 RightDark -30 LeftBright -30 RightBright -30 LeftDark 30 RightDark 30 LeftBright 30 RightBright 30 LeftDark 60 RightDark 60 LeftBright 60 RightBright 60 motionerror

Figure S1: Saccade kinematics and identification of oculomotor-tuned ROIs. Related to Figure 1.

(A) Distributions of post-saccadic eye position, amplitude and peak velocity for convergent and conjugate adducting saccades. Median $\pm$ IQR for $N = 76$ animals, with spline fits. (B) Overview of analysis of saccade-related activity. First, saccade-active ROIs were selected based on their saccade-triggered activity modulation (see C). Second, ridge regression was used to identify oculomotor-tuned ROIs (see D). Each oculomotor-tuned ROI was classified as $Conv$, $ConjL/R$ or $Both$ according to the saccade type(s) for which it was active (see F). (C) For each ROI, d' values were computed for each saccade type and compared to null distributions calculated by shuffling saccade onset times 1000-fold. When d' exceeded the 95th percentile of the shuffle distribution, the ROI was considered active for the corresponding saccade type. ROIs active for at least one saccade type were considered saccade-active. (D) Ridge regression was used to model the fluorescence time-series (zF) of each saccade-active ROI as a linear function of sensory and motor regressors. The unique contribution of each regressor to the model was quantified by circularly permuting it and assessing the fractional change in cross-validated goodness-of-fit (ΔcvR^2). By repeating the process for every regressor, a vector of ΔcvR^2 values is generated for each ROI. Finally, ROIs were classified as oculomotor-tuned when an oculomotor regressor produced the largest decrement in model performance (most negative ΔcvR^2). For further details, see Methods. (E) Example ridge regression fits for two ROIs. The box shows recorded and model-predicted fluorescence as well as eye position, tail curvature and saccade and stimulus times during a portion of the experiment. *Right:* All 33 predictors are shown (prior to convolution with a CIRF, see Methods) for a larger portion of the experiment. Portion in the box is highlighted. (F) Venn diagrams showing the proportion of ROIs active for each saccade type, with classification key. (G) ΔcvR^2 vectors for oculomotor-tuned ROIs (left) and all other saccade-active ROIs (right). ROIs have been ordered by the regressor with most negative ΔcvR^2.

Midbrain and hindbrain distribution of oculomotor-tuned ROIs

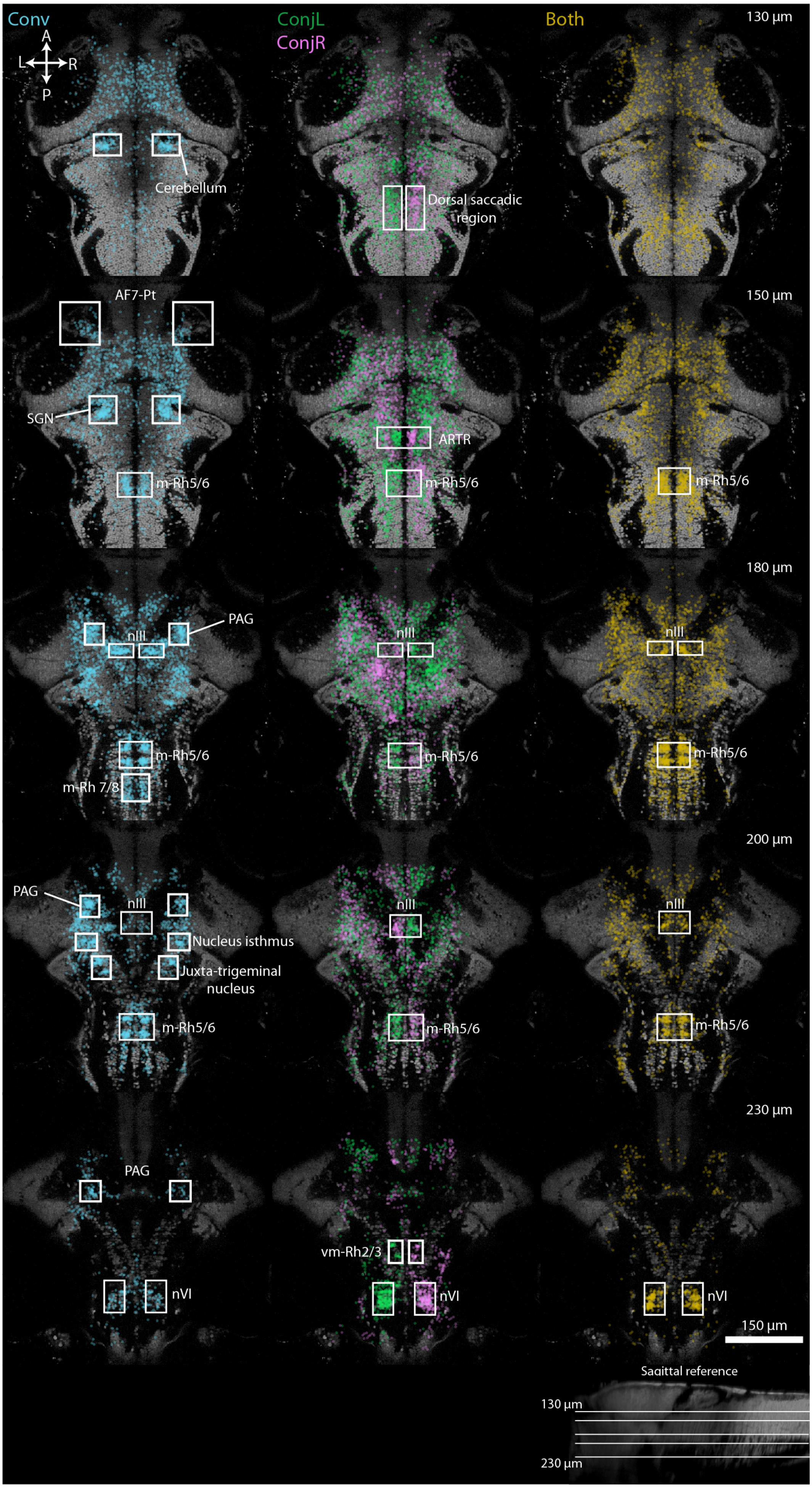

Figure S2: Oculomotor-tuned neurons. Related to Figure 1.

Oculomotor-tuned ROIs active for convergent (*Conv*) or leftwards/rightwards conjugate (*ConjL/R*) or both (*Both*) saccade types shown in ZBB reference brain space (44,332 neurons from 76 animals). Panels show horizontal planes at the dorsoventral location indicated in the top right corner and shown on a sagittal view at the bottom of the figure. All three types of oculomotor-tuned cell are found in the oculomotor and abducens nuclei as well as medial rhombomere-5/6 (m-Rh5/6), close to the facial motor nucleus. In addition, *Conj* ROIs, predominantly with ipsiversive tuning, are abundant in dorsal rhombomere 5–7, where eye velocity-related activity has been described[S1,S2],the anterior rhombencephalic turning region (ARTR)[S3], and in ventromedial rhombomere 2/3 (vm-Rh2/3), adjacent to reticulospinal neurons. *Conv* ROIs are found in regions previously implicated in hunting, including the pretectum adjacent to retinal arborization field 7 (AF7-Pt)[S4] and the nucleus isthmus[S5]. In addition, a high density are observed in the secondary gustatory nucleus (SGN)[S6], the dorso-medial cerebellum, medial rhombomere 7/8 (m-Rh7/8), a tegmental region likely corresponding to the periaqueductal grey (PAG), and in close proximity to the trigeminal motor nuclei (juxta-trigeminal region). *Abbreviations*: nIII, oculomotor nucleus; nVI, abducens nucleus; ARTR, anterior rhombencepahlic turning region; SGN, secondary gustatory nucleus; vm-Rh2/3, ventro-medial rhombomere 2/3; m-Rh5/6, medial rhombomere 5/6; m-Rh7/8, medial rhombomere 7/8; PAG, periaqueductal grey.

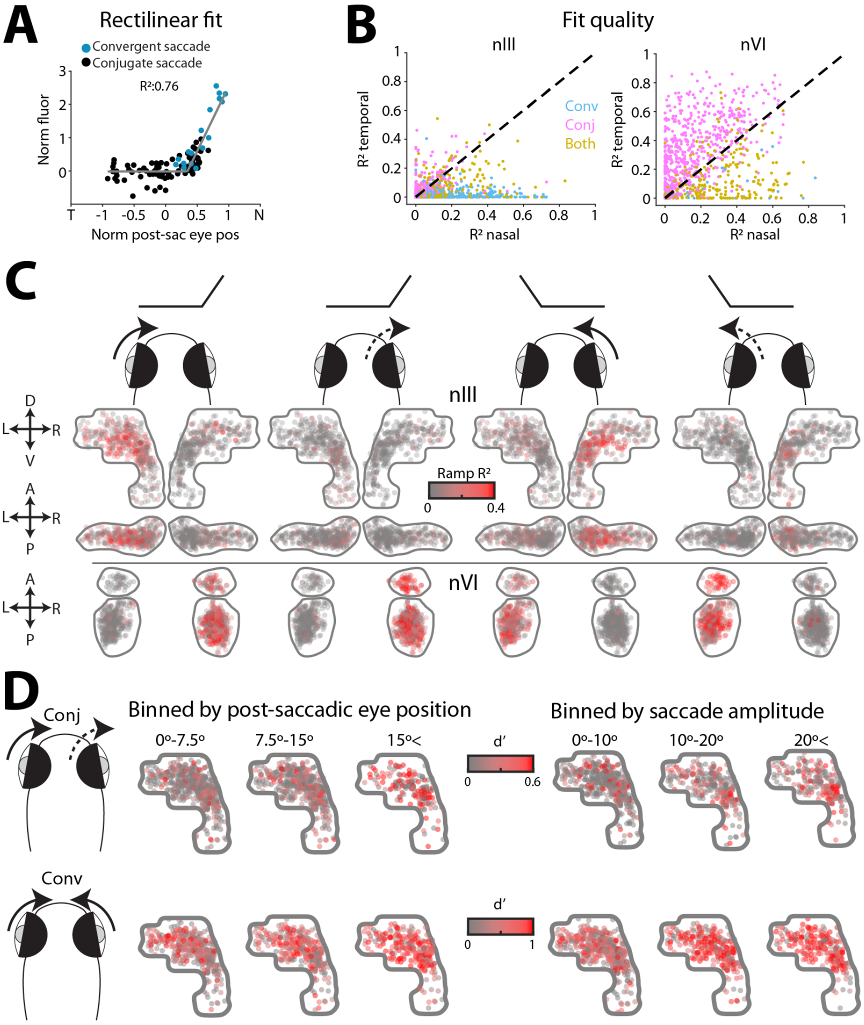

Figure S3: Functional properties in oculomotor and abducens nuclei. Related to Figure 2.

(A) Rectilinear fit of saccade-triggered change in fluorescence as a function of post-saccadic eye position, for an example neuron. (B) Comparison of R^2 for rectilinear fits for nasal versus temporal eye movements in the 'preferred direction' of each ROI (see Methods). (C) Oculomotor-tuned ROIs colour-coded by R^2 for rectilinear fits for each eye–direction contingency. From left to right: left eye nasal, right eye temporal, right eye nasal, left eye temporal. (D) Oculomotor-tuned ROIs coloured by saccade-triggered activity (d') for conjugate and convergent saccades binned by post-saccadic eye position (left), or amplitude (right).

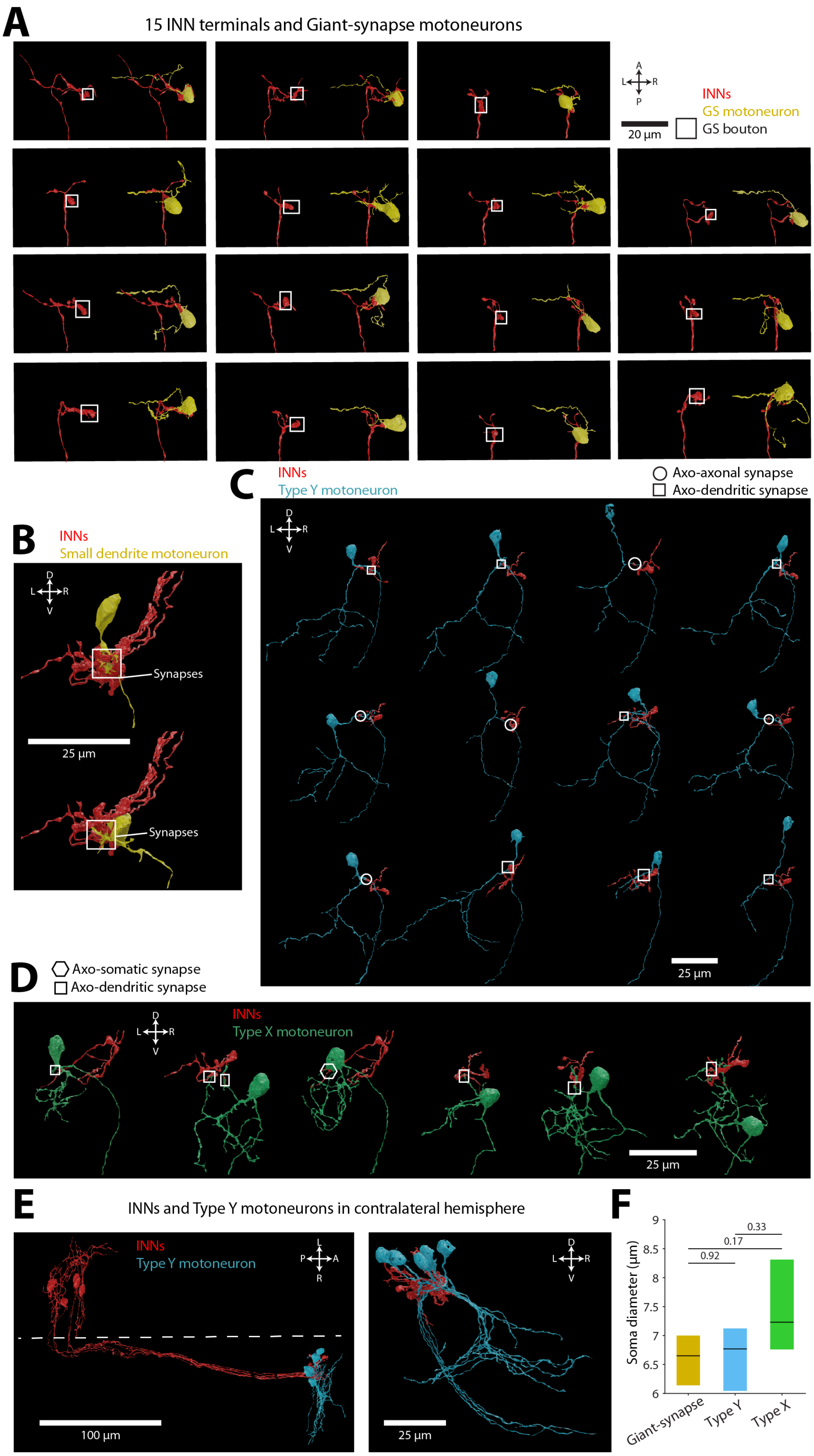
A
15 INN terminals and Giant-synapse motoneurons
INNs
GS motoneuron
GS bouton
20 µm
B
INNs
Small dendrite motoneuron
Synapses
Synapses
25 µm
C
INNs
Type Y motoneuron
Axo-axonal synapse
Axo-dendritic synapse
25 µm
D
Axo-somatic synapse
Axo-dendritic synapse
INNs
Type X motoneuron
25 µm
E
INNs and Type Y motoneurons in contralateral hemisphere
INNs
Type Y motoneuron
100 µm
25 µm
F
Soma diameter (µm)
0.92
0.17
0.33
Giant-synapse
Type Y
Type X

Figure S4: Ultrastructural reconstructions of individual motoneurons and pre-synaptic INN axon terminals. Related to Figure 3.

(A) 3D reconstructions of 15 giant-synapse motoneurons (yellow) and pre-synaptic INN terminals (red). Terminals forming the giant synapse indicated by white boxes. (B) 3D reconstructions of two motoneurons that formed synapses with multiple INN boutons on claw-like dendrites. (C–D) 3D reconstructions of 12 Type Y motoneurons (C) and 6 Type X motoneurons (D). (E) 3D reconstructions of an additional 6 INNs and 5 Type Y motoneurons, traced from the contralateral hemisphere. (F) Soma diameters for different motoneuron types (median (IQR) across $N = 15$ giant-synapse, 12 Type Y, 6 Type X). Kruskal-Wallis with Tukey-Kramer post-hoc tests.

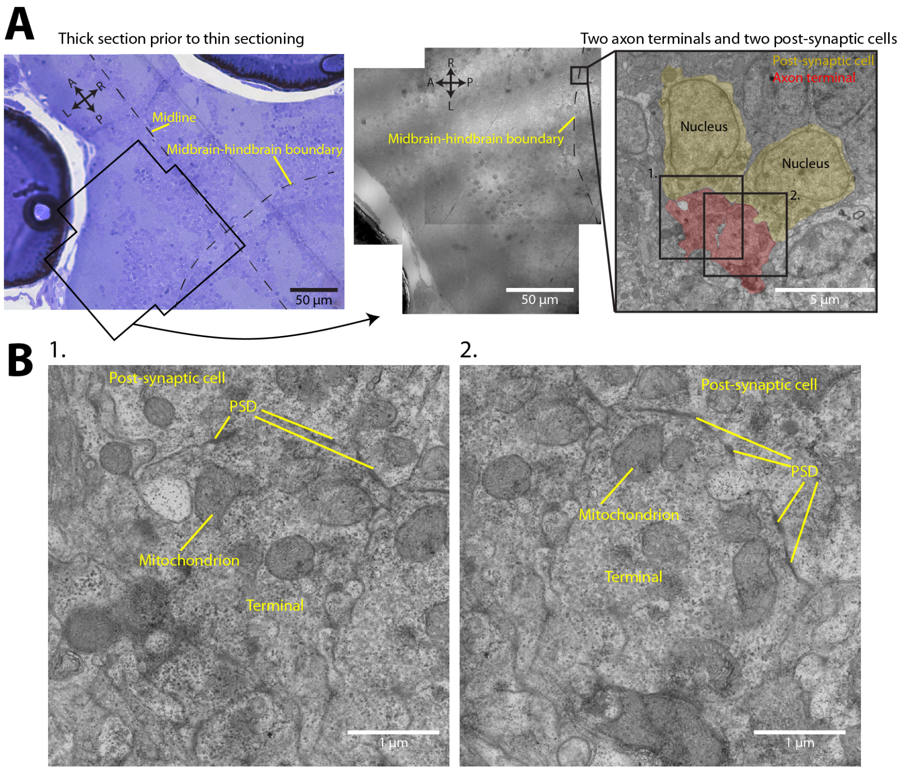

Figure S5: Transmission electron micrographs of giant synapses in oculomotor nucleus. Related to Figure 3.

(A) *Left:* Toluidine blue-stained thick (2 μm) horizontal section used to guide thin sectioning for electron microscopy. Electron micrograph area shown by black outline. *Middle:* Three electron micrographs encompassing the midbrain-hindbrain boundary, aligned and overlayed. *Right:* High-magnification electron micrograph of region outlined in middle panel. Two giant axo-somatic synaptic appositions are highlighted. Boxes indicate extent of electron micrographs in B. (B) Higher magnification electron micrographs of the two giant synapses. Multiple post-synaptic densities (PSDs) can be seen at the post-synaptic membrane.

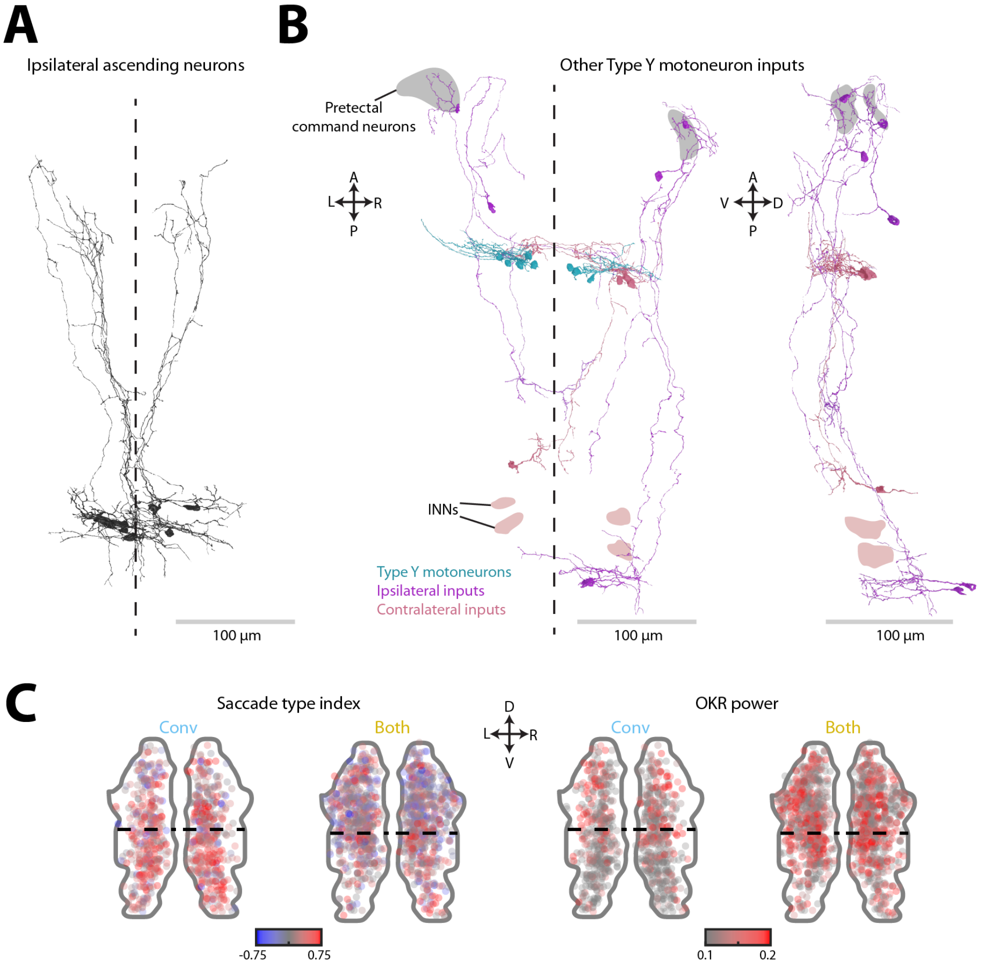

Figure S6: Other inputs to Type Y motoneurons and functional metrics in m-Rh5/6. Related to Figure 5.

(A) Ultrastructural reconstructions of nine m-Rh5/6 neurons that extended ipsilateral ascending projections to the caudal midbrain. (B) Neurons identified as presynaptic to Type Y motoneurons, other than those with somata in m-Rh5/6. Recipient Type Y motoneurons shown in the horizontal projection (left). Areas corresponding to the soma locations of INNs and pretectal command neurons are highlighted. (C) Maps of oculomotor-tuned ROIs in m-Rh5/6 colour-coded by functional metrics.

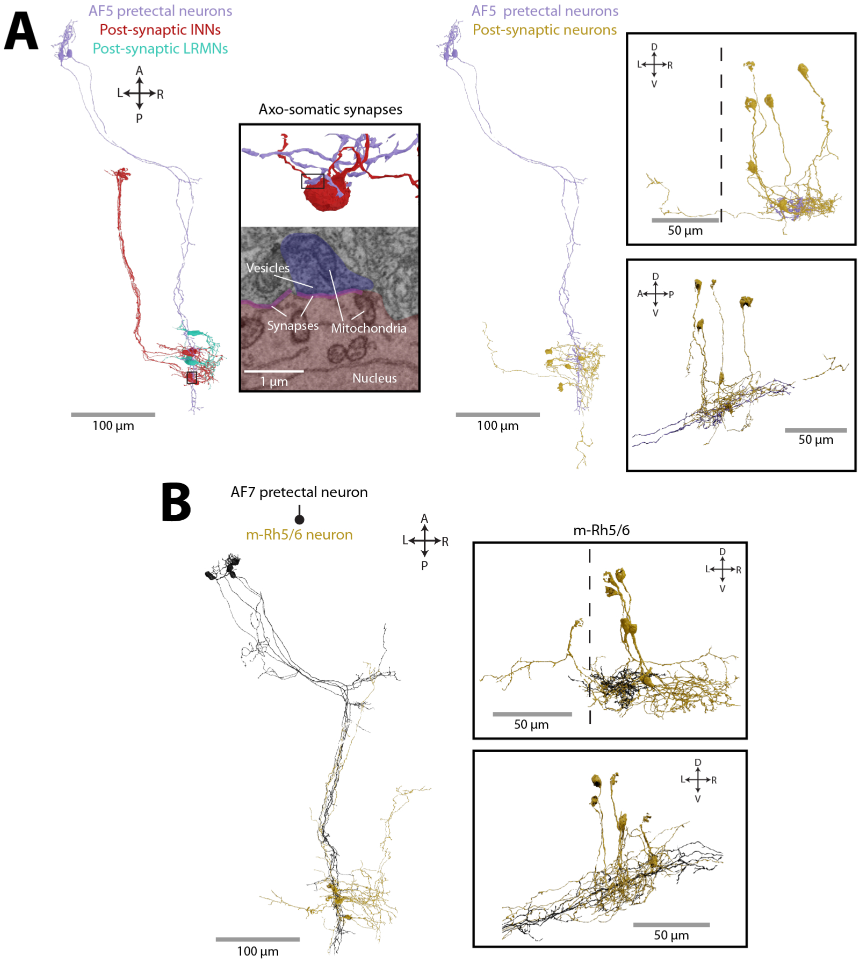

Figure S7: Pretectal projection neurons – additional data. Related to Figure 7.

(A) *Left:* Horizontal projection of two AF5-pretectal neurons with seven post-synaptic INNs and five LRMNs. Inset box shows a close-up 3D reconstruction and electron micrograph of an axo-somatic synapse onto an INN. *Right:* Horizontal projection of the same AF5-pretectal neurons along with six post-synaptic neurons in m-Rh5/6. Inset boxes show coronal (top) and sagittal (bottom) views of m-Rh5/6 region. (B) Horizontal projection of AF7-pretectal neurons and post-synaptic cells in m-Rh5/6. Inset boxes show coronal (top) and sagittal (bottom) views of m-Rh5/6.

Predictor name	Description	Type	Continuous/one-hot encoding/binary vector	Convolved with CIRF
ConvGL	Convergent saccade with leftwards post-saccadic version	Oculomotor	one-hot	Yes
ConvGR	Convergent saccade with rightwards post-saccadic version	Oculomotor	one-hot	Yes
LConj	Left conjugate saccade	Oculomotor	one-hot	Yes
RConj	Right conjugate saccae	Oculomotor	one-hot	Yes
Lc	Contraversive left eye position	Oculomotor	continuous	Yes
Rc	Contraversive right eye position	Oculomotor	continuous	Yes
LVigQ1	Leftwards swim in the first quartile of swim vigour	Locomotor	one-hot	Yes
LVigQ2	Leftwards swim in the second quartile of swim vigour	Locomotor	one-hot	Yes
LVigQ3	Leftwards swim in the third quartile of swim vigour	Locomotor	one-hot	Yes
LVigQ4	Leftwards swim in the fourth quartile of swim vigour	Locomotor	one-hot	Yes
RVigQ1	Rightwards swim in the first quartile of swim vigour	Locomotor	one-hot	Yes
RVigQ2	Rightwards swim in the second quartile of swim vigour	Locomotor	one-hot	Yes
RVigQ3	Rightwards swim in the third quartile of swim vigour	Locomotor	one-hot	Yes
RVigQ4	Rightwards swim in the fourth quartile of swim vigour	Locomotor	one-hot	Yes
OKRL	Leftwards optokinetic drifting grating	Stimulus	binary vector	Yes
OKRR	Leftwards optokinetic drifting grating	Stimulus	binary vector	Yes
LeftDark -60	Prey-like moving spot (leftwards, dark) at -60 deg azimuth	Stimulus	one-hot	Yes
RightDark -60	Prey-like moving spot (rightwards, dark) at -60 deg azimuth	Stimulus	one-hot	Yes
LeftBright -60	Prey-like moving spot (leftwards, bright) at -60 deg azimuth	Stimulus	one-hot	Yes
RightBright -60	Prey-like moving spot (rightwards, bright) at -60 deg azimuth	Stimulus	one-hot	Yes
LeftDark -30	Prey-like moving spot (leftwards, dark) at -30 deg azimuth	Stimulus	one-hot	Yes
RightDark -30	Prey-like moving spot (rightwards, dark) at -30 deg azimuth	Stimulus	one-hot	Yes
LeftBright -30	Prey-like moving spot (leftwards, bright) at -30 deg azimuth	Stimulus	one-hot	Yes
RightBright -30	Prey-like moving spot (rightwards, bright) at -30 deg azimuth	Stimulus	one-hot	Yes
LeftDark 30	Prey-like moving spot (leftwards, dark) at 30 deg azimuth	Stimulus	one-hot	Yes
RightDark 30	Prey-like moving spot (rightwards, dark) at 30 deg azimuth	Stimulus	one-hot	Yes
LeftBright 30	Prey-like moving spot (leftwards, bright) at 30 deg azimuth	Stimulus	one-hot	Yes
RightBright 30	Prey-like moving spot (rightwards, bright) at 30 deg azimuth	Stimulus	one-hot	Yes
LeftDark 60	Prey-like moving spot (leftwards, dark) at 60 deg azimuth	Stimulus	one-hot	Yes
RightDark 60	Prey-like moving spot (rightwards, dark) at 60 deg azimuth	Stimulus	one-hot	Yes
LeftBright 60	Prey-like moving spot (leftwards, bright) at 60 deg azimuth	Stimulus	one-hot	Yes
RightBright 60	Prey-like moving spot (rightwards, bright) at 60 deg azimuth	Stimulus	one-hot	Yes
motionerror	Magnitude of motion correction transformation	Motion error	continuous	No

Table S1: List of regressors used for linear modelling. Related to Figure 1.

Supplemental References

S1. Wolf, S., Dubreuil, A.M., Bertoni, T., Böhm, U.L., Bormuth, V., Candelier, R., Karpenko, S., Hildebrand, D.G.C., Bianco, I.H., Monasson, R., et al. (2017). Sensorimotor computation underlying phototaxis in zebrafish. Nat Commun *8*, 651.

S2. Leyden, C., Brysch, C., and Arrenberg, A.B. (2021). A distributed saccade-associated network encodes high velocity conjugate and monocular eye movements in the zebrafish hindbrain. Sci Rep *11*, 12644.

S3. Dunn, T.W., Mu, Y., Narayan, S., Randlett, O., Naumann, E.A., Yang, C.T., Schier, A.F., Freeman, J., Engert, F., and Ahrens, M.B. (2016). Brain-wide mapping of neural activity controlling zebrafish exploratory locomotion. Elife *5*, e12741.

S4. Antinucci, P., Folgueira, M., and Bianco, I.H. (2019). Pretectal neurons control hunting behaviour. Elife *8*.

S5. Henriques, P.M., Rahman, N., Jackson, S.E., and Bianco, I.H. (2019). Nucleus Isthmi Is Required to Sustain Target Pursuit during Visually Guided Prey-Catching. Curr Biol *29*, 1771–1786.e5.

S6. Shainer, I., Kuehn, E., Laurell, E., Al Kassar, M., Mokayes, N., Sherman, S., Larsch, J., Kunst, M., and Baier, H. (2023). A single-cell resolution gene expression atlas of the larval zebrafish brain. Sci Adv *9*, eade9909.
